# Muscle parameters in fragility fracture risk prediction in older adults: A scoping review

**DOI:** 10.1002/jcsm.13418

**Published:** 2024-01-29

**Authors:** Colin Vendrami, Enisa Shevroja, Elena Gonzalez Rodriguez, Guillaume Gatineau, Jolanda Elmers, Jean‐Yves Reginster, Nicholas C. Harvey, Olivier Lamy, Didier Hans

**Affiliations:** ^1^ Interdisciplinary Center of Bone Diseases, Rheumatology Unit, Department of Bone and Joint Lausanne University Hospital and University of Lausanne Lausanne Switzerland; ^2^ Internal Medicine Unit, Department of Internal Medicine Lausanne University Hospital and University of Lausanne Lausanne Switzerland; ^3^ University Library of Medicine, Faculty of Biology and Medicine Lausanne University Hospital and University of Lausanne Lausanne Switzerland; ^4^ WHO Collaborating Center for Public Health Aspects of Musculo‐Skeletal Health and Ageing, Division of Public Health, Epidemiology and Health Economics University of Liège Liège Belgium; ^5^ MRC Lifecourse Epidemiology Centre University of Southampton Southampton UK

**Keywords:** fragility fracture, frailty, muscle, older adults, osteoporosis, risk, sarcopenia

## Abstract

Half of osteoporotic fractures occur in patients with normal/osteopenic bone density or at intermediate or low estimated risk. Muscle measures have been shown to contribute to fracture risk independently of bone mineral density. The objectives were to review the measurements of muscle health (muscle mass/quantity/quality, strength and function) and their association with incident fragility fractures and to summarize their use in clinical practice. This scoping review follows the PRISMA‐ScR guidelines for reporting. Our search strategy covered the three overreaching concepts of ‘fragility fractures’, ‘muscle health assessment’ and ‘risk’. We retrieved 14 745 references from Medline Ovid SP, EMBASE, Web of Science Core Collection and Google Scholar. We included original and prospective studies on community‐dwelling adults aged over 50 years that analysed an association between at least one muscle parameter and incident fragility fractures. We systematically extracted 17 items from each study, including methodology, general characteristics and results. Data were summarized in tables and graphically presented in adjusted forest plots. Sixty‐seven articles fulfilled the inclusion criteria. In total, we studied 60 muscle parameters or indexes and 322 fracture risk ratios over 2.8 million person‐years (MPY). The median (interquartile range) sample size was 1642 (921–5756), age 69.2 (63.5–73.6) years, follow‐up 10.0 (4.4–12.0) years and number of incident fragility fractures 166 (88–277). A lower muscle mass was positively/not/negatively associated with incident fragility fracture in 28 (2.0), 64 (2.5) and 10 (0.2 MPY) analyses. A lower muscle strength was positively/not/negatively associated with fractures in 53 (1.3), 57 (1.7 MPY) and 0 analyses. A lower muscle function was positively/not/negatively associated in 63 (1.9), 45 (1.0 MPY) and 0 analyses. An in‐depth analysis shows how each single muscle parameter was associated with each fragility fractures subtype. This review summarizes markers of muscle health and their association with fragility fractures. Measures of muscle strength and function appeared to perform better for fracture risk prediction. Of these, hand grip strength and gait speed are likely to be the most practical measures for inclusion in clinical practice, as in the evaluation of sarcopenia or in further fracture risk assessment scores. Measures of muscle mass did not appear to predict fragility fractures and might benefit from further research, on D3‐creatine dilution test, lean mass indexes and artificial intelligence methods.

## Introduction

Osteoporosis is characterized by a generalized loss of bone mass and altered microarchitecture, leading to an increased risk of fracture.^1^ Over the age of 50, a fifth of men and half women will have a fragility (or osteoporotic) fracture, developed spontaneously or after a minor trauma, such as a fall from a standing height.^1^ Major osteoporotic fractures (MOFs) include hip, vertebral, humeral and forearm fractures. Fragility fractures are a major age‐related adverse event due to their consequences and high incidence.^2^ Osteoporotic fractures account for more days of hospitalization than acute myocardial infarction, chronic obstructive pulmonary disease or breast cancer.^3^ In Europe, the direct costs were estimated at 37.4 billion euros in 2010 and 56.9 billion euros in 2019^2^ and will continue to increase as the population aged over 65 and over 80 is expected to double and triple respectively between 2020 and 2050.^4^ Bone fragility can be prevented and treated. However, the gap in its management consists in the limited capacities to detect and predict fragility fractures.^5^

The gold standard for assessing bone mineral density (BMD) is dual‐energy X‐ray absorptiometry (DXA). The World Health Organization (WHO) defines osteoporosis as a BMD of 2.5 standard deviations below the mean peak BMD of young female adults.^6^ However, half of fractures occurs in individuals with a normal BMD.^7^ Risk scores have thus been developed and have improved fracture prediction, by taking into consideration other clinical risk factors for fractures^8^; the most widely used fracture risk score is FRAX® (Fracture Risk Assessment Tool).^8^ Although FRAX with BMD performs better than BMD alone in predicting incident fractures, there is still room for improvement in risk prediction, potentially through inclusion of additional measures, such as falls, that are independent of BMD.^9^ Muscles lose 40% of their volume between the ages of 20 and 80.^10^ Since the first mention of the muscles mass loss as sarcopenia by Rosenberg in 1989,^11^ many parameters of muscle health have been studied using a variety of measures such as radiological imaging, strength measurements, functional assessments and blood tests. In parallel, the definition of sarcopenia has evolved to a composite loss of muscle mass, strength and function, and its association with adverse outcomes, including fragility fractures.^12^ Sarcopenia and osteoporosis are both associated with ageing and similar risk factors in a close interaction.^13^ They increase the risk of falls, fragility fractures, surgery, chronic pain, physical disability, social isolation and death.^14^, ^15^, ^16^, ^17^, ^18^, ^19^ All these negative consequences lead to higher hospital costs and longer hospital stays.^20^, ^21^, ^22^

A scoping review is a structured approach to summarize and map the evidence and gaps on a topic. This type of knowledge synthesis is particularly useful for planning future research on heterogeneous and broad topics. So far, only one scoping review studied muscle health and its association with adverse outcomes.^23^ The authors focused on three definitions of sarcopenia and their ability to predict various adverse outcomes. Of the 11 included studies in this previous review, only one analysed fragility fractures.^24^ The currently available studies on muscle health parameters and their association with incident fragility fractures have not been fully reviewed.

The objectives of this scoping review were (1) to review muscle health assessment techniques (muscle mass/quantity/quality, strength and function) and their association with incident fragility fractures and (2) to summarize the clinical use of the parameters associated with fragility fractures risk.

## Methodology

This scoping review followed the Preferred Reporting Items for Systematic Reviews and Meta‐Analyses extension for Scoping Review (PRISMA‐ScR) guidelines for reporting and the JBI methodology for writing.^25^, ^26^ The PRISMA‐ScR checklist is provided in the supporting information. The study protocol is available online in the OSF (Open Science Framework) registry at https://archive.org/details/osf‐registrations‐2fmtg‐v1 (registration DOI: 10.17605/OSF.IO/2FMTG).

### Inclusion criteria

The studies included in this review fulfilled the following criteria: (1) original study; (2) participants over 50 years of age recruited from the general population (community‐dwelling) without gender, racial, geographic or cultural restriction. Studies where the participants were recruited on the basis of a medical condition (e.g., frailty, osteoporosis and cancer) were excluded to minimize selection bias; (3) assessment of at least one muscle health parameter; (4) prospective studies; (5) fragility fracture as outcome: a low‐trauma fracture at any specific osteoporotic site or a combination of sites; and (6) the association of each muscle health parameter with the fragility fracture incidence was examined. No language restrictions were performed. Meta‐analyses, systematic reviews and, text/opinion papers relevant to the current review's question were considered for the qualitative and critical evaluation and interpretation.

### Source of evidence and search strategy

A systematic search strategy was developed with a research librarian to cover the three overarching concepts of the research: ‘fragility fractures’, ‘muscle health assessment’ and ‘risk’. The search syntax contains free and index/mesh terms, a filter to exclude animal studies and a general filter for the study types. Relevant articles were also compared to better define the keywords and index terms of the equations. The search strategy was translated for the following databases: Medline Ovid SP, EMBASE and Web of Science Core Collection. A complementary search equation was developed for Google Scholar. Systematic search syntaxes are available in the supporting information. Unpublished studies and grey literature were not screened. Backward and forward citation chasing of eligible studies was also done. We also undertook hand searching of references within records and on specific authors to identify further eligible studies. The search included article published from inception of the databases to 27 April 2023.

### Study selection

**Figure 1:**
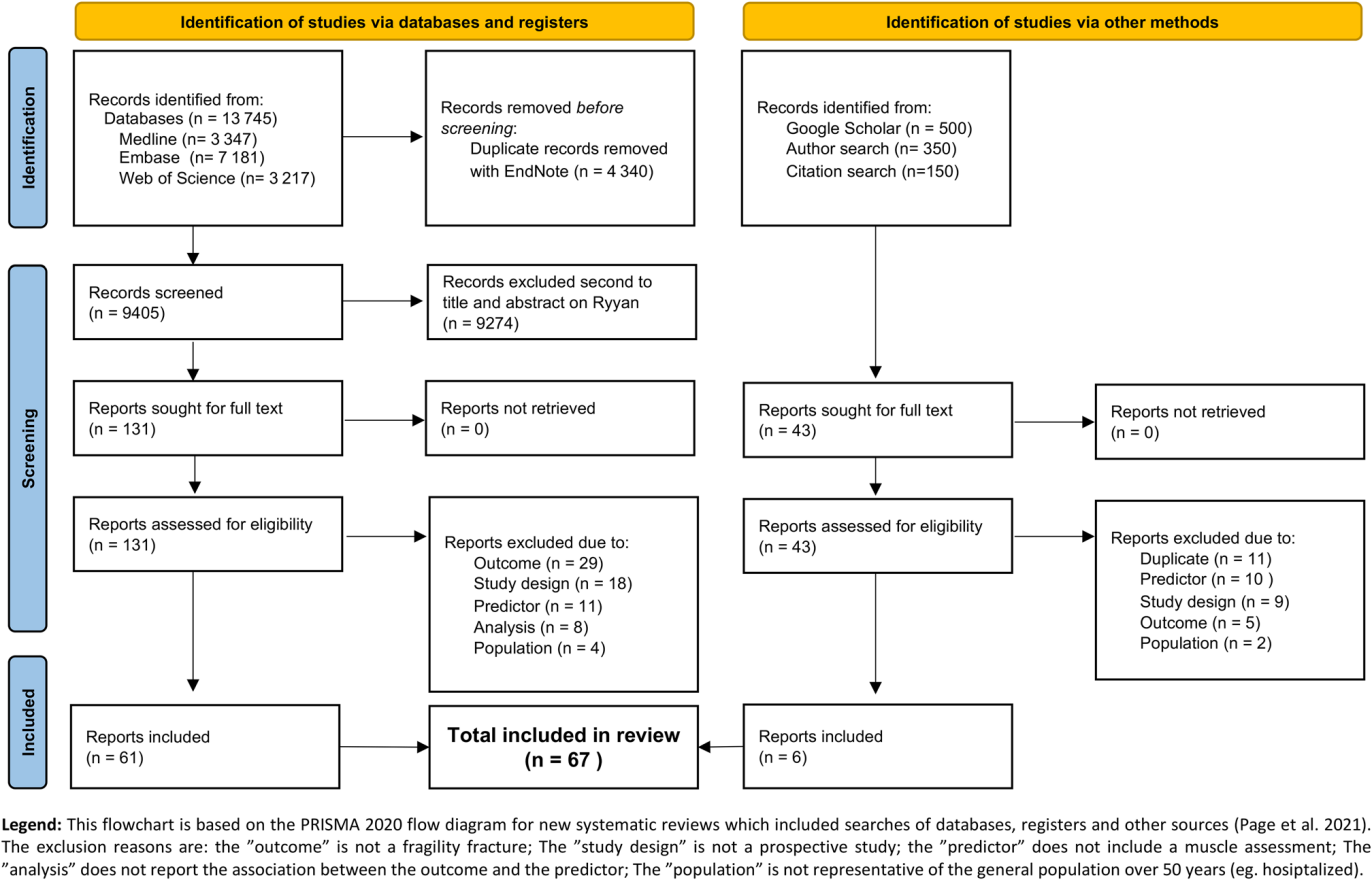
PRISMA 2020 flow diagram of the study.

The identified citations from the systematic search were de‐duplicated (J. E.) in EndNote™ (Clarivate Analytics, Philadelphia, PA, USA) and transferred (C. V.) to Rayyan (*free web application for systematic reviews* ^27^). One author (C. V.) screened the titles and abstracts for eligibility and retrieved the full texts of the selected articles. The reasons for exclusion were recorded at full text reading. The study's selection process is fully reported using the PRISMA 2020 flow diagram (cf. *Figure* *1*).

### Data extraction and qualitative assessment

The data were extracted from the included articles by one author (C. V.) using an Excel table. For each study, qualitative and quantitative data were extracted^25^: first author, year of publication, country, design, duration of follow‐up, population, sex, mean age at baseline, sample size, muscle health parameter, fracture type, number of fractures, statistical approach, model adjustments and fracture risk estimates for the muscle parameters studied. When one association had multiple models, we kept the model considering the strongest predictor of fragility fractures including age and/or BMD. Multiple adapted forest plots were used to visually demonstrate the overall trends of associations between each muscle parameter and the fracture risk. The results were grouped by mass/quantity/quality (*Figures* *3*, *4*, *5*), strength and function and by fracture type (A–F). The results were ordered by parameter, measure subtype, sex and publication date. The muscle mass mostly refers to lean mass (LM) (or its estimation) while quantity also includes volumes and areas. Muscle quality is a broad terminology and includes muscle density, muscle texture, myosteatosis, muscle fat infiltration and some ultrasound measures.^28^ In order to homogenize the reporting and to facilitate the interpretation of the results, we always reported the fracture risk ratios for a lower/slower/deteriorated muscle parameter (e.g., ‘the risk ratio for 1 SD decrease in lean mass’). Most of the original articles had reported the fracture risk ratio per unit of deterioration in the muscle parameter studied, and these values were reported identically; if the original article had reported the fracture risk ratios per increase in the muscle parameter studied, we calculated and reported the 1/risk ratio. The rationale is that a worsened/unhealthy muscle parameter is associated with a higher risk of fracture. Finally, the most frequently cited muscle health assessment parameters in the included articles are briefly discussed in terms of their generalizability and availability in clinical practice.^29^ Additionally, the best predictors of fragility fractures are reported, including the total person‐year.

## Results

### Characteristics of the included studies

**Figure 2:**
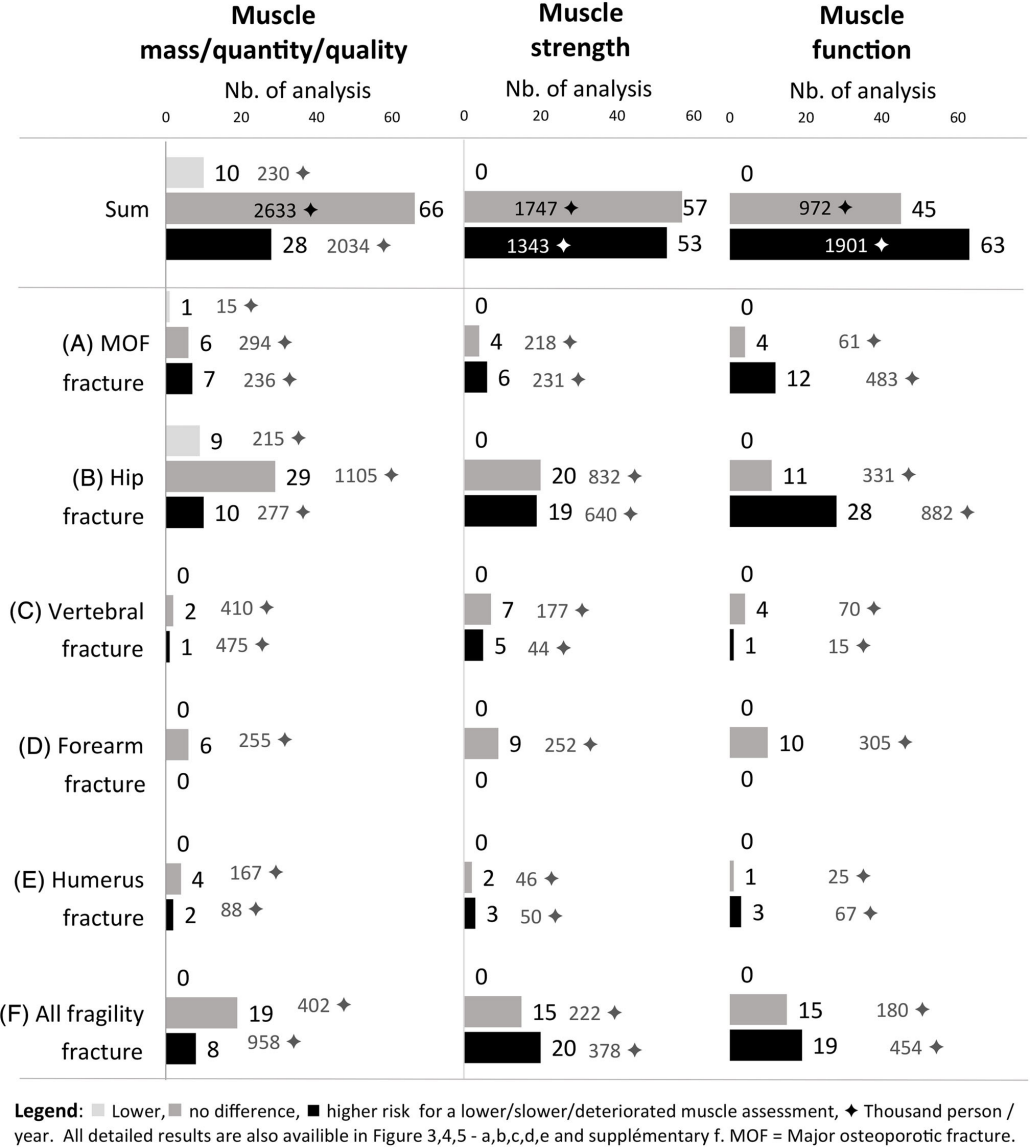
(A–F) Summary of the 322 analyses for each muscle assessment and each fracture types.

**Figure 3:**
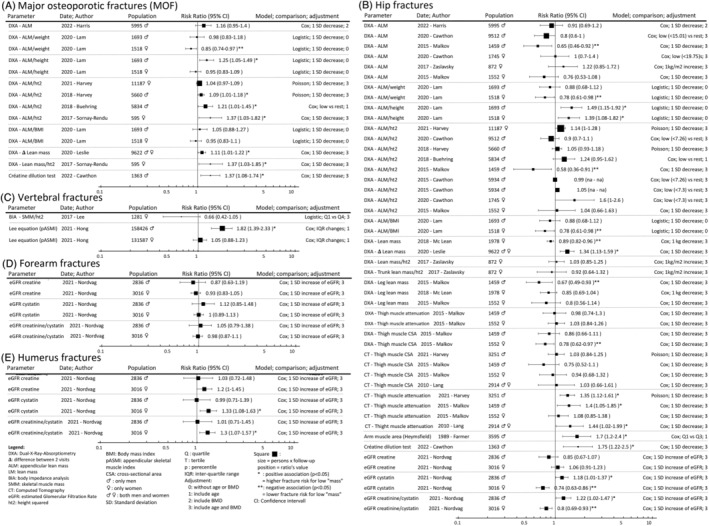
(A–E) Muscle mass/quantity/quality parameters and risk of incident fragility fractures.

**Figure 4:**
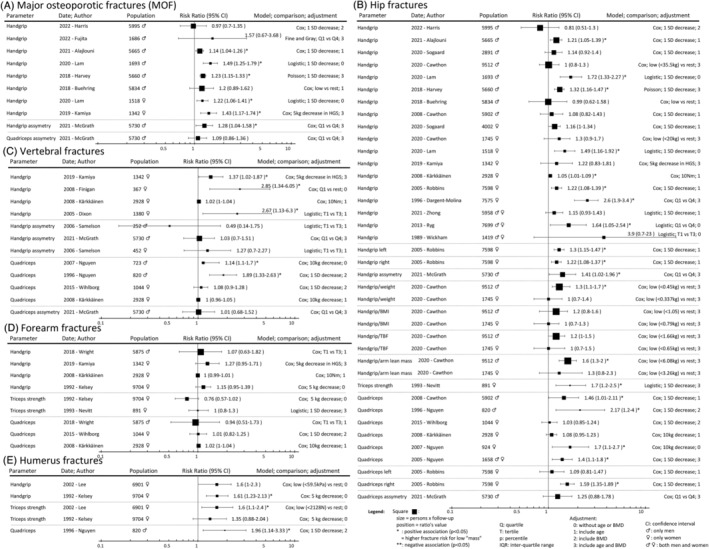
(A–E) Muscle strength parameters and risk of incident fragility fractures.

**Figure 5:**
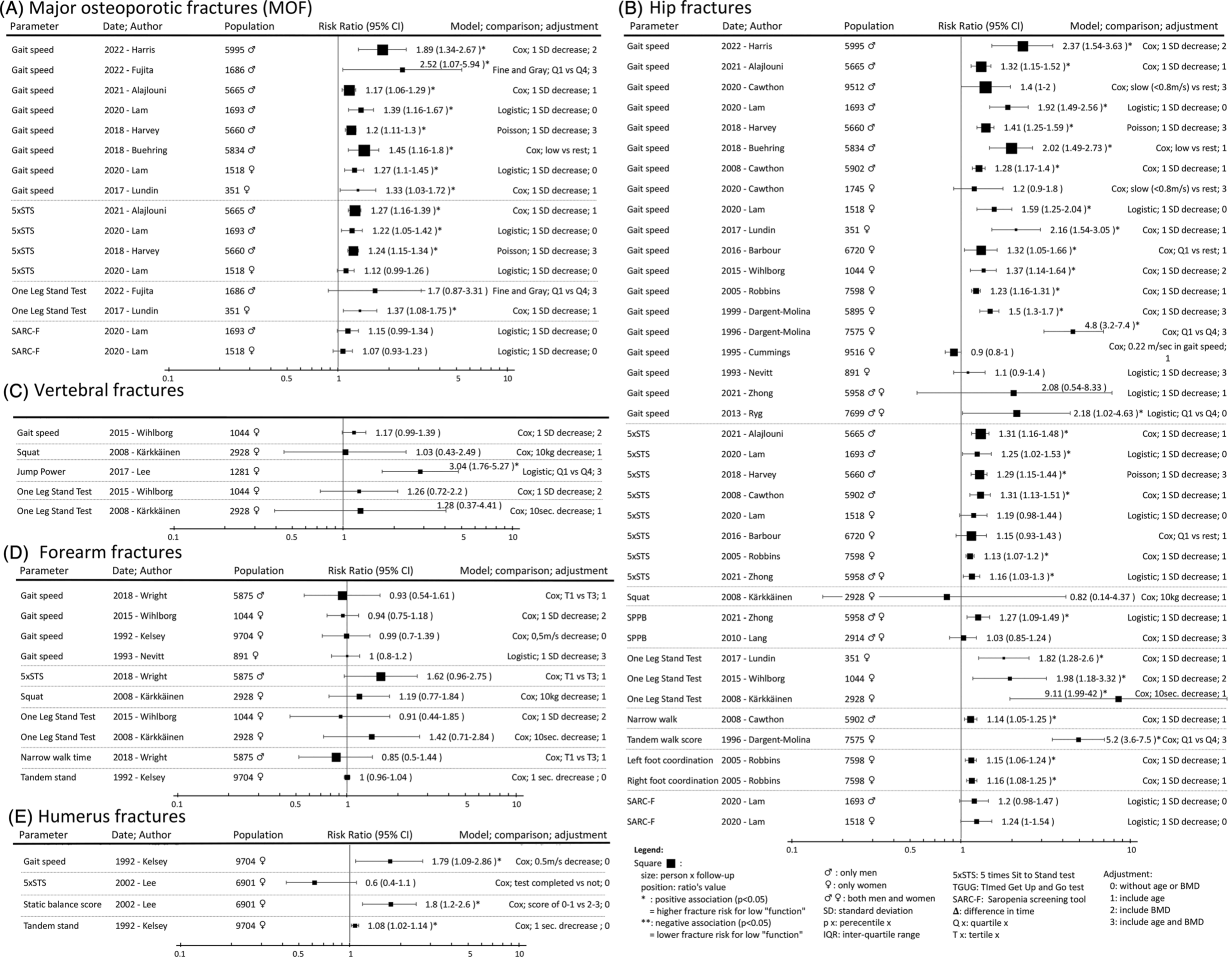
(A–E) Muscle function parameters and risk of incident fragility fractures.

Of the 13 745 studies extracted from the databases and the approximately 1000 studies screened using additional methods (*Figure* *1*: PRISMA flow chart), 67 studies were included in this review, comprising 2.8 million person‐years: median sample size (1st–3rd quartile) of 1642 (921–5756) participants, follow‐up of 10.0 (4.4–12.0) years, age of 69.2 (63.5–73.6) years and number of incident fragility fractures of 166 (88–277).^30^, ^31^, ^32^, ^33^, ^34^, ^35^, ^36^, ^37^, ^38^, ^39^, ^40^, ^41^, ^42^, ^43^, ^44^, ^45^, ^46^, ^47^, ^48^, ^49^, ^50^, ^51^, ^52^, ^53^, ^54^, ^55^, ^56^, ^57^, ^58^, ^59^, ^60^, ^61^, ^62^, ^63^, ^64^, ^65^, ^66^, ^67^, ^68^, ^69^, ^70^, ^71^, ^72^, ^73^, ^74^, ^75^, ^76^, ^77^, ^78^, ^79^, ^80^, ^81^, ^82^, ^83^, ^84^, ^85^, ^86^, ^87^, ^88^, ^89^, ^90^, ^91^, ^92^, ^93^, ^94^, ^95^, ^96^ The general characteristics of the included studies are summarized in *Table* *1* and detailed for each article in *Table* *2*. The most cited cohorts were MrOS (USA, China and Sweden; 13 articles), DOES (Australia; 6 articles), SOF (USA; 5 articles), Health ABC (USA; 4 articles) and EPIDOS (France; 4 articles). Within the studies, 37 analysed women, 30 men and 13 both together. All results and references are presented visually and summarized in multiple stacked plots (*Figures* *3*, *4*, *5*). The 67 included studies investigated 60 different muscle parameters and were grouped into 6 types of fragility fracture: hip (*Figure* *2* *B*: 126 analyses), all type of fragility fractures (*Figure* *2* *F*: 96 analyses), MOF (*Figure* *2* *A*: 40 analyses), forearm (*Figure* *2* *D*: 25 analyses), vertebral (*Figure* *2* *C*: 20 analyses) and humerus (*Figure* *2* *E*: 15 analyses), for a total of 322 analyses. The studies used different statistical approaches such as logistic, Cox proportional, Poisson or Fine and Gray models and different adjustments (*Table* *2* and *Figures* *3*, *4*, *5*: ‘Model; comparison; adjustment’). The following three sections summarize the main results for each muscle characteristic: mass and quantity (*Figures* *3* *A–E* and *S3f*), strength (*Figures* *4* *A–E* and *S4f*) and function (*Figures* *5* *A–E* and *S5f*).

**Table 1:** Summary of the 67 included studies and main characteristics

Most cited first authors (nb. of articles)	Cawthon (5), Nguyen (4), Harvey (3)
Years of publications	From 1989 to 2022, most in 2020
Most cited cohorts (nb. of articles)	MrOS (13), DOES (6), SOF (5), Health ABC (4), EPIDOS (4)
Most represented country (nb. of articles)	USA (22), Australia (8), China (6), Sweden (6), France (6)
Study design	Prospective only
Median follow‐up (years)	10.0 (IQR: 4.4–12.0)
Most studied population	Community‐dwelling healthy older adults
Sex sub‐groups in the analysis (M/W)	Women = 37, men = 30, both (and adjusted for sex) = 13
Median age (years)	69.2 (IQR: 63.5–73.6)
Median sample size	1642 (IQR: 921–5756)
Most analysed parameter (nb. of analysis)	Hand grip strength (76), gait speed (49), DXA–ALMI (28), quadriceps strength (28), chair rising tests (27)
Most studied fragility fractures (nb. of analysis)	Hip (126), all fragility fx (96), MOF (40), forearm (25), vertebral (20), humerus (15), total (322)
Median incident fractures per study	166 (IQR: 88–277)
Most used statistical methods	Hazard ratio and 95% confidence interval, for 1 standard deviation worsening/degradation of the muscle parameter
Most used adjustment factors	Age, weight, height, BMD and sex

*Note*: Chair rising tests include the timed up and go test and the five‐time sit‐to‐stand test. Abbreviations: BMD, bone mineral density; DXA–ALMI, appendicular lean mass index/height^2^ from dual‐energy X‐ray absorptiometry; IQR, interquartile range; MOF, major osteoporotic fracture.

**Table 2:** Characteristics of included studies

^Ref^Author Date	Study name or city (country)	Follow‐up (years)	Population inclusion	Sex	Age (years)	Sample size	Predictors	Fragility fracture type	Nb. of fractures	Statistical test	Statistical comparison	Selected adjustments/covariables
^30^Yamada 2022	Maibara city (Japan)	3.0	Community‐dwelling over 65 years, recruited through email	♂♀	73.8 ± 6.0	773	US	All fall‐related fractures	51	Cox proportional HR	T1 vs. T3 (95% CI)	Age, sex, BMI, cognitive function and polypharmacy
^31^Harris 2022	MrOS (USA)	12.0	Ambulatory community‐dwelling over 65 years	♂	73.7 ± 5.9	5995	HGS GS DXA	All fragility MOF Hip	1414	Cox proportional HR	1 SD decrease (95% CI)	BMD T‐score, history of diabetes, history of arthritis/gout history of falls, self‐reported health rating, depressive feelings, PASE score, smoking status, alcohol per week, living alone, education status, visual acuity, use of benzodiazepines, use of selective serotonin reuptake inhibitors and GS score
^32^Fujita 2022	FORMEN (Japan)	8.4	Community‐dwelling able to walk, consent and self‐report information recruited through printed literature	♂	73.1 ± 5.2	1686	GS OLST HGS	All fragility MOF Hip	175	Fine and Gray subdistribution HR	Q1 vs. Q4 (95% CI)	Age, BMI, BMD, drinking habits (≥1 day/week), smoking habits, history of type 2 diabetes mellitus, history of prostate cancer with hormone therapy, history of gastrectomy and history of falls at baseline study visit
^33^Cawthon 2022	MrOS (USA)	4.6	Ambulatory community‐dwelling over 65 years	♂	84.2	1363	D3Cr dilution test	MOF Hip	180	Cox proportional HR	1 SD decrease (95% CI)	Age, falls, FRAX® and BMD
^41^Alajlouni 2021	MrOS (USA)	12.7	Ambulatory community‐dwelling over 65 years	♂	73.5 ± 5.8	5665	GS HGS 5×STS	MOF Hip	1014	Cox proportional HR	1 SD decrease (95% CI)	Garvan and FRAX® parameters
^34^Zhong 2021	CHARLS (China)	4.0	Representative sample over 60 years living in households	♂♀	67.5 ± 6.7	5958	SPPB	Hip	180	Logistic regression	1 SD decrease (95% CI)	Age, gender, body mass index, education level, falls and chronic diseases (including diabetes, chronic lung diseases, kidney disease, arthritis or rheumatism)
^40^Harvey 2021	WHI (USA)	14.1	Postmenopausal women from 50 to 79 years at baseline	♀	63.3 ± 7.4	11 187	ALM ALM/height^2^	MOF Hip	1225	Poisson regression	1 SD decrease (95% CI)	Age, follow‐up time and FRAX**®** + BMD
^37^Harvey 2021	MrOS (USA, Sweden and China)	7.4	Ambulatory community‐dwelling over 65 years	♂	76.0 ± 5.3	3251	pQCT	Hip	112	Poisson regression	1 SD decrease (95% CI)	Falls, FRAX**®** and femoral neck BMD
^39^Hong 2021	NHIS‐HEALS (Korea)	3.0	National representative random sample	♀	60.7 ± 8.4	131 587	Lee equation (pASMI)	All Vertebral	6175	Cox proportional HR	IQR changes (95% CI)	Age, income, physical activity, smoking, alcohol consumption, systolic blood pressure, fasting serum glucose, total cholesterol, Charlson Comorbidity Index and body mass index
				♂	60.2 ± 8.3	158 426			2350			
^36^Nordvåg 2021	Tromsø Study (Norway)	14.6	All inhabitant over 50 years that accepted to participate	♀	63.5 ± 6.3	3016	Creatinine, cystatin, creatinine/cystatin (as eGFR)	Hip Wrist Humerus	761	Cox proportional HR	1 SD decrease of creatinine (increase of eGFR) (95% CI)	Age, height, BMI, BMD, smoking, history of previous fracture and diabetes, high‐sensitivity C‐reactive protein and use of corticosteroid and any blood pressure‐lowering drugs
		14.6		♂	62.8 ± 6.5	2836			218			Age, height, BMI, BMD, smoking, history of previous fracture, diabetes and cardiovascular disease, and use of any blood pressure‐lowering drugs
^38^McGrath 2021	MrOS (USA)	8.7	Ambulatory community‐dwelling over 65 years	♂	73.6 ± 5.9	5730	HGS symmetry QS symmetry	MOF Hip Clinical spine	438	Cox proportional HR	Q1 vs. Q4 of asymmetry (95% CI)	Baseline maximum leg extension power or maximum hand grip strength (for the appropriate predictor), age, clinic site, race, alcohol intake, cigarette smoking status, body mass index, cognitive functioning, physical activity participation, morbidities, benzodiazepine usage and femoral neck bone mineral density
^35^Westbury 2021	Health ABC (USA)	10.0	Random selection of White, and all Black, from 70 to 79 years without physical disability	♂♀	74.0 ± 2.9	2480	HGS GS ALM 𝚫 ALM	All	401	Fine and Gray subdistribution HR	1 SD decrease (95% CI)	Height, weight‐for‐height residual, smoking status (ever vs. never), alcohol consumption, healthy eating index, physical activity, educational attainment, home ownership, cognitive function and number of comorbidities
^45^Cawthon 2021	SDOC (USA, Sweden, China and Australia)	8.9	Community‐dwelling over 65 years	♀	≥65	1745	GS HGS DXA	Hip	166	Cox proportional HR	Binary outcomes (95% CI)	Age, self‐rated health, pain, use of statins, cognitive function, cancer, congestive heart failure, stroke, chronic obstructive pulmonary disease and diabetes, plus bone mineral density for hip fracture models and competing risk of death
		10.2		♂		9512			392			
^46^Alajlouni 2020	DOES2 W (Australia)	18.0	Community‐dwelling over 60 years	♀	68.6 ± 4.2	811	TGUG 5×STS GS HGS QS ALMI	All	224	Cox proportional HR	Q1 vs. Q2–Q4 (95% CI)	Age, femoral neck BMD, prior fractures, falls, BMI, smoking, alcohol, physical activity, diabetes, neurological diseases, cardiovascular diseases, cancer, hypertension, respiratory diseases and renal failure
		18.0		♂	69.2 ± 3.8	440			74			
^43^Leslie 2020	Manitoba (USA)	6.0	DXA record	♂♀	67.0 ± 10.0	9622	TBLM	MOF Hip	692	Cox proportional HR	1 SD decrease (95% CI)	FRAX**®** with BMD, including competing mortality
^42^Søgaard 2020	Tromsø Study (Norway)	15.0	All inhabitant over 50 years that accepted to participate	♀	61.0 ± 7.4	4002	HGS	All Hip	868	Cox proportional HR	1 SD decrease (95% CI)	Age, height, BMI, marital status, level of education, leisure time physical activity, daily smoking, consumption of alcohol, self‐perceived health and self‐reported one or more diseases
		15.0		♂	62.9 ± 6.5	2891			231			
^44^Lam 2020	MrOS (China)	10.0	Community‐dwelling recruited through notices, stratified by age	♀	72.6 ± 5.4	1518	SARC‐F GS HGS 5×STS ALM + indexes	MOF Hip	236	Logistic regression	1 SD decrease (95% CI)	Univariate
		10.0		♂	72.4 ± 5.0	1693			139			
^47^Scott 2019	CHAMP (Australia)	6.0	Over 70 years from electoral roll of New South Wales	♂	76.7 ± 5.4	1575	HGS GS ALM/height	All	63	Logistic regression	1 SD decrease (95% CI)	Age, income, living alone, number of comorbidities, smoking status, psychotropic and corticosteroid use, history of fracture, physical activity and 25(OH)D
^48^Kamiya 2019	JPOS (Japana)	15.2	Over 50 years randomly selected from resident registration	♀	63.4 ± 8.5	1342	HGS	All Hip	162	Cox proportional HR	5‐kg decrease in HGS (95% CI)	Age, BMD, previous vertebral/hip fracture and BMI
^49^Cronholm 2019	MrOS (Sweden)	9.6	Community‐dwelling able to walk, from the register of 3 cities	♂	75.4 ± 3.2	3014	HGS	All	683	Cox proportional HR	1 SD decrease (95% CI)	Univariate
^54^Harvey 2018	MrOS (USA, Sweden and China)	10.0	Ambulatory community‐dwelling	♂	73.5 ± 10.9	5660	ALM/height^2^ HGS 5×STS	All MOF Hip fracture	14–35%	Fine and Gray subdistribution HR	1 SD decrease (95% CI)	FRAX**®** + BMD
^51^Schaap 2018	LASA (Netherland)	10.0	Population registries of 11 municipalities, stratified by age (over 65 years) and sex	♂	75.2 ± 6.4	498	HGS GS DXA	All	60	Cox proportional HR	Low (EWGSOP1) vs. others (95% CI)	Age, sex and total body fat
^55^Buehring 2018	MrOS (USA)	14.0	Ambulatory community‐dwelling	♂	74 ± 6	5834	HGS GS ALM/height^2^	MOF Hip	635	Cox proportional HR	Low vs. others (95% CI)	Age, falls, osteoporosis, body fat, muscle mass, grip strength and gait speed
^53^Kim 2018	Ansung (Korea)	1.0	Community‐dwelling	♀	63.3 ± 8.6	1627	HGS DXA	All	156	Logistic regression	Low AWG1 vs. rest (95% CI)	Age, osteoporosis, total fat mass, current smoking, regular exercise, comorbidity and osteoporosis medication
				♂	62.9 ± 8.5	1201			56			
^52^McLean 2018	Framingham (USA)	8.3	Over 50 years with DXA	♀	66.9	1978	DXA leg DXA total body	Hip	99	Cox proportional HR	1‐kg decrease (95% CI)	Age, height, study cohort, per cent total body fat, femoral neck BMD, history of hip fracture, smoking, physical activity, oestrogen replacement use and osteoporosis medication use
^50^Wright 2018	MrOS (USA)	10.8	Ambulatory community‐dwelling	♂	65.0–69.0	5875	HGS 5×STS Leg power Narrow walk GS	Wrist	97	Cox proportional HR	T1 vs. T3 (95% CI)	Age, race/ethnicity and study site
^60^Harris 2017	WHI (USA)	15.9	Healthy postmenopausal women from 40 centres	♀	63.3 ± 0.07	10 973	DXA	All	1648	Cox proportional HR	Low Newman mass vs. others (95% CI)	Age, race, study assignment, physical function, history of fracture, history of self‐report falls in the past year, hormone use, physical activity, alcohol consumption, smoking status, corticosteroid use, BMI, dietary calcium intake and dietary vitamin D intake
^57^Sornay‐Rendu 2017	OFELY (France)	13.1	Volunteers randomly selected from insurance company	♀	66.0 ± 8.0	595	DXA	All MOF	138	Cox proportional HR	1 SD decrease (95% CI)	Age, previous fracture, femoral neck BMD, physical activity, incident falls and risk of death
^58^Lundin 2017	PRIMO (Sweden)	10.0	Born in 1920–1930 in Bagarmossen contacted	♀	73.0	351	GS OLST	MOF Hip	40	Cox proportional HR	1 SD decrease (95% CI)	Age
^59^Lee 2017	KURE (KOREA)	12.0	Over 65 years selected through recruiters, poster promotion, health visit, self‐acquaintance	♀	71.0 ± 4.4	1281	BIA Jump power	Vertebral	282	Logistic regression	Q1 vs. Q4 (95% CI)	Age, BMD, serum 25(OH)D level, body fat percentage, previous fracture, parental hip fracture, alcohol, smoking, physical activity, grip strength, cognitive impairment and weight loss over the past year
^56^Zaslavsky 2017	WHI (USA)	11.5	Over 65 years with ≥3 Fried's criteria	♀	72.3 ± 4.52	872	DXA total and regional lean and fat	Hip fracture	49	Cox proportional HR	1 kg/m^2^ increase (95% CI)	Age, ethnicity, smoking, history of previous fractures, recurrent falls and several frailty criteria, and BMD
^61^Balogun 2017	TASOAC (Australia)	10.0	Over 50 years, sex stratified from an electoral roll	♂♀	63.0 ± 7.5	1041	HGS DXA Lower limb strength	All		Poisson regression	‘Low’ vs. others (95% CI)	Age
^63^Hars 2016	GERICO (Switzerland)	3.4	Retirees	♂♀	65.0 ± 1.4	913	DXA	All	40	Logistic regression	Low EWGSOP or IWG vs. others (95% CI)	Gender, age, length of follow‐up and FRAX**®** probability with femoral neck BMD
^64^Barbour 2016	SOF (USA)	9.0	From US clinics	♀	70.4	6720	GS 5×STS	Hip	266	Cox proportional HR	Q1 vs. Q2–Q4 (95% CI)	Age at enrolment, interaction between age and PF_age80, physical performance trajectory, interaction between age and physical performance trajectory, BMI, walk for exercise, smoking, alcohol use, calcium use, oestrogen use, health status, falls in the past 12 months, prevalent fracture after age 50 years, stroke, hypertension, diabetes, cognitive function and hip BMD
^66^Malkov 2015	Health ABC (USA)	13.5	Random White and all Black from 70 to 79 years without physical disability	♀	70.0–79.0	1552	CT DXA	Hip	105	Cox proportional HR	1 SD decrease (95% CI)	Age, race, clinical site, BMI, chronic disease, hip BMD, self‐reported health, alcohol use, smoking status, education, physical activity and cognitive function
				♂		1459			64			
^62^Pham 2016	DOES (Australia)	11.0	Community‐dwelling over 60 years	♀	68.9 ± 5.0	1066	HGS	All	289	Cox proportional HR	1 SD decrease (95% CI)	Femoral neck BMD, age and prior fracture, history of fall and smoking
		11.0		♂	69.7 ± 5.0	595			89			
^67^Cawthon 2015	MrOS (USA)	9.8	Community‐dwelling over 65 years	♂	73.6 ± 6.0	5934	DXA Newman equation	Hip	207	Cox proportional HR	Change in C‐statistic compared with adjusted model only (95% CI)	Age and BMD
^65^Wihlborg 2015	OPRA (Sweden)	10.0	Random selection with 75 years	♀	77.7 ± 0.2	1044	Balance GS QS	Hip Vertebral All	427	Cox proportional HR	1 SD decrease (95% CI)	History of fracture, BMI, smoking habits, bisphosphonate, vitamin D, glucocorticoid and alcohol use
^68^Yu 2014	MrOS (China)	11.3	Community‐dwelling recruited through notices, stratified by age	♂	65.0	2000	DXA, GS	All	226	Cox proportional HR	Low AWG1 vs. rest (95% CI)	Age, education levels, socio‐economic status ladder, presence of chronic obstructive pulmonary disease, diabetes mellitus, hypertension, heart diseases and stroke, smoking, physical activity (PASE total score), dietary protein intake, dietary vitamin D intake, dietary energy intake, cognitive function (CSI‐D categories), and body weight and hip BMD
^69^Ryg 2013	SHARE (Europe)	4.0	n.a.	♂♀	63.3	7699	HGS, GS	Hip	216	Logistic regression	Q1 vs. Q4 (95% CI)	Body mass index, country and falls
^71^Edwards 2012	Hertfordshire (UK)	5.5	n.a.	♀	66.2 ± 2.8	1418	HGS	All	n.a.	Logistic regression	1‐kg decrease (95% CI)	Age, height, weight‐adjusted‐for‐height, social class, smoking status, alcohol consumption, activity score and dietary calcium
		5.5		♂		1579						
^70^Rouzi 2012	(Saudi Arabia)	5.2	Postmenopausal women over 50 years from multistage random sampling	♀	61.3 ± 7.2	707	HGS, TUG, GS, 5×STS	All	148	Logistic regression	Q1 vs. Q4 (95% CI)	Univariate
^72^Cheung 2012	Hong Kong (China)	2.9	Recruited from public roadshows and health fairs	♂♀	64.1 ± 9.5	1702	HGS	All (clinical)	43	Cox proportional HR	1 SD decrease (95% CI)	Age, sex, BMI, history of fall, diabetes, current smoking, current drinking, physical activity (exercise > 1 h/week), presence of prevalent fracture and femoral neck BMD T‐score
^73^Lang 2010	Health ABC (USA)	6.6	Random White and all Black from 70 to 79 years without physical disability	♂♀	73.5 ± 2.8	2914	CT QS SPPB	Hip	63	Cox proportional HR	1 SD decrease (95% CI)	Age, height, BMI, total percentage of fat, race, gender, clinical site and BMD
^74^Sirola 2008	OSTPRE (Finland)	15.0	Random stratified sample from postal enquiry to women	♀	53.3 ± 2.9	971	HGS	All	271	Cox proportional HR	Q1 vs. Q4 (95% CI)	Fracture history, body mass index, age, years since menopause, use of hormonal replacement therapy, alcohol intake, smoking, nutritional calcium intake and bone‐affecting diseases/medications
^75^Kärkkäinen 2008	OSTPRE (Finland)	8.4	Random stratified sample from postal enquiry to women	♀	59.1 ± 2.9	2928	HGS QS OLST Squatt	Hip, Vertebral, Forearm	261	Cox proportional HR	10‐Nm HGS, 10‐kg decrease quadriceps, 10 s. OLST (95% CI)	Age, BMI, current smoking, years since menopause, years of hormonal therapy and history of fracture
^76^Finigan 2008	Sheffield (UK)	10.0	Random selection from general practitioner list in Sheffield stratified by age	♀	64.6 ± 9.1	367	HGS	Vertebral	99	Cox proportional HR	Q1 vs. Q2–Q4 (95% CI)	Univariate
^77^Cawthon 2008	MrOS (USA)	5.3	Over 65 years	♂	73.4	5902	HGS QS GS Narrow walk 5×STS	Hip	77	Cox proportional HR	1 SD decrease (95% CI)	Age, clinical centre, femoral neck bone mineral density, body mass index, history of heart attack and history of stroke
^78^Nguyen 2007	DOES (Australia)	15.0	Community‐dwelling over 60 years	♀	69.0 ± 6.3	924	QS	Hip	221	Cox proportional HR	10‐kg decrease (95% CI)	Univariate
				♂	69.7 ± 6.0	723		Clinical vertebral	105			
^79^Sipilä 2006	Evergreen Project (Finland)	10.0	All from one city aged 75 years	♀	75.0	187	Knee strength Elbow strength	Hip (fall related)	n.a.	Cox proportional HR	n.a. (95% CI)	Height and BMD
^80^Shigematsu 2006	Evergreen Project (Finland)	10.0	All participants between 75 and 80 years from one city that accepted	♂♀	78.0 ± 0.1	307	QS Motor speed and reaction	All (fall related)	94	Cox proportional HR	T1 vs. T3 (95% CI)	Age, sex and BMD
^81^Samelson 2006	Framingham (USA)	25.0	Random selection from Framingham city	♀	54.0	452	HGS	Vertebral	110	Logistic regression	T1 vs. T3 (95% CI)	Age, height, weight, prevalent vertebral fracture, smoking and alcohol consumption
				♂		252			25			
^82^Pluijm 2006	LASA (Netherland)	3.0	Stratified sample of 55–85 years from 11 municipalities in Netherland	♂♀	75.3 ± 6.4	1365	HGS	All (fall related)	87	Cox proportional HR	Quintile 1 vs. rest (95% CI)	Univariate
^83^Robbins 2005	EPIDOS (France)	3.0	Volunteers selected from voters or health registers from 5 French areas	♀	80.5	7598	HGS QS GS 5×STS coordination	Hip	293	Cox proportional HR	1 SD decrease (95% CI)	Age only, but results also stratified by BMD class
^84^Nguyen 2005	DOES (Australia)	12.0	All over 60 years from Dubbo	♂♀	70.6 ± 7.2	1658	QS	Hip	115	Cox proportional HR	1 SD decrease (95% CI)	Gender, age and femoral neck BMD
^85^Dixon 2005	EPOS (Europe)	3.8	Population registers across Europe	♀	63.6 ± 8.2	1380	HGS	Vertebral	34	Logistic regression	T1 vs. T3 (95% CI)	Age, BMI, lifetime activity score and current activity
^86^Albrand 2003	OFELY (France)	5.3	Postmenopausal women, stratified by age, randomly selected from health insurance company	♀	59.1 ± 9.8	672	Left HGS GS Tandem balance Tandem walking speed Chair stand	All	81	Logistic regression	Group median difference (95% CI)	Univariate, except for grip strength
^87^Lee 2002	EPIDOS (France)	3.6	Volunteers selected from voters or health registers from 5 French areas	♀	80.5 ± 3.7	6901	HGS Triceps strength 5×STS Static balance	Proximal humerus	165	Cox proportional HR	Low vs. high (95% CI)	Univariate
^88^Dargent‐Molina 1999	EPIDOS (France)	2.8	Volunteers selected from voter or health registers from 5 French areas	♀	80.5 ± 3.8	5895	GS	Hip	170	Cox proportional HR	1 SD decrease (95% CI)	Age, femoral BMD and calcaneal broadband ultrasound attenuation
^90^Dargent‐Molina 1996	EPIDOS (France)	1.9	Volunteers selected from voter or health registers from 5 French areas	♀	80.5 ± 3.8	7575	HGS GS Calf circumference Tandem walk	Hip	154	Cox proportional HR	Q1 vs. Q4 (95% CI)	Age, centre, calf circumference, gait speed, tandem walk score, visual acuity and BMD
^89^Nguyen 1996	DOES (Australia)	5.0	Community‐dwelling over 65 years	♂	n.a.	820	QS	All	166	Cox proportional HR	1 SD decrease (95% CI)	BMD
^91^Cummings 1995	SOF (USA)	4.1	White, over 65 years and able to walk	♀	72.0 ± 5.0	9516	GS	Hip	192	Logistic regression	0.22 m/s in gait speed (95% CI)	Age and ability to raise from a chair
^93^Nevitt 1993	SOF (USA)	4.1	Non‐Black, aged over 65 years, living in the community	♀	72.2 ± 5.6	891	Triceps strength GS	Hip Wrist	424	Logistic regression	1 SD decrease (95% CI)	For the other covariates used as predictors, age and radius BMD
^92^Nguyen 1993	DOES (Australia)	3.0	All over 60 years from Dubbo	♀	69.2 ± 6.6	1080	QS	All fragility fracture	104	Logistic regression	0.45 SD decrease (95% CI)	Univariate
				♂	69.0 ± 6.3	709			38			
^94^Kelsey 1992	SOF (USA)	2.2	White, over 65 years and able to walk	♀	65.0–79.0	9704	Balance GS HGS Triceps strength	Humerus Distal forearm	250	Cox proportional HR	5‐kg decrease for HGS and triceps, 0.5 m/s decrease for GS, 1 s for tandem stand (95% CI)	Univariate
^95^Wickham 1989	DHSS (UK)	15.0	Community‐dwelling over 65 years	♂♀	65.0–74.0	1419	HGS	Hip	44	Logistic regression	T1 vs. T3 between hip fracture and match (95% CI)	BMI and smoking
^96^Farmer 1989	NHANES I (USA)	10	White	♀	40–77	3595	Arm muscle area	Hip	84	Cox proportional HR	Q1 vs. Q3 (95% CI)	Age, recreational activity, activity apart from recreation, menopausal status, smoking and calcium

Abbreviations: 5×STS, five‐time sit‐to‐stand test; ALM, appendicular lean mass; BIA, body impedance analysis; BMD, bone mineral density; BMI, body mass index; CI, confidence interval; CT, computed tomography; DXA, dual‐energy X‐ray absorptiometry; eGFR, estimated glomerular filtration rate; EWGSOP, European Working Group on Sarcopenia in Older People; FRAX®, Fracture Risk Assessment Tool; GS, gait speed; HGS, hand grip strength; HR, hazard ratio; IQR, interquartile range; IWG, International Working Group on Sarcopenia; MOF, major osteoporotic fracture (hip, spine, forearm or humerus); n.a., not applicable; OLST, one‐leg standing test; pASMI, predicted appendicular skeletal muscle index; pQCT, peripheral quantitative CT; QS, quadriceps strength; SARC‐F, sarcopenia questionnaire; SD, standard deviation; SPPB, Short Physical Performance Battery test; TBLM, total body lean mass; TGUG, timed get up and go test; US, ultrasound. *Source*: Characteristics extraction adapted from Peters et al.^97^

### Muscle mass, quantity and quality

Evaluation of muscle mass and quantity has been performed by very different methods, from radiological images (i.e., DXA and computed tomography [CT]), biological measures (creatine dilution test) or even anthropometric prediction equations. Globally, a lower muscle mass or quantity was associated with risk of incident fragility fracture in 28 (2034 thousand person‐years [TPY]) analyses, no risk in 66 (2633 TPY) analyses and lower risk in 10 (230 TPY) analyses (*Figures* *2*, *3* *A–E* and *S3f*). Body composition analysis by DXA was the most used method. Several DXA‐derived muscle mass parameters were analysed: appendicular lean mass (ALM), change in ALM, ALM/height, ALM/height^2^, change in ALM/height^2^, ALM/weight, ALM/body mass index (BMI), total LM, change in total LM, total LM/height^2^, regional LM, thigh muscle cross‐sectional area and thigh muscle attenuation. A lower DXA‐derived muscle mass parameter was associated with a higher, no and a lower fragility fracture risk in 15 (408 TPY), 46 (1609 TPY) and 8 (145 TPY) analyses, respectively. A lower ALM/height^2^ was associated with a higher, no and a lower fragility fracture risk in 5 (158 TPY), 22 (997 TPY) and 1 (20 TPY) analyses, respectively. However, when considering MOF only, lower ALM/height^2^ was associated with a higher and no fracture risk in three (147 TPY) and one (158 TPY) studies. Of the MOF subtypes, only the hip fractures were studied with DXA‐derived parameters; namely, ALM/height^2^ was negatively associated in one (20 TPY) study, and there was no association in eight (547 TPY) studies. No study analysed the association between lower ALM/height^2^ and incident vertebral, forearm and humeral fracture. The bioelectrical impedance analysis (BIA) was not associated with vertebral fractures in one (15 TPY) analysis using skeletal muscle mass/height^2^. The ultrasonography of the quadriceps (US) was not associated with fragility fractures in one (2 TPY) analysis using quadriceps quantity/quality. The parameters derived from the CT scan (lower thigh muscle cross‐sectional area representing muscle mass and lower thigh muscle attenuation representing muscle quality) were positively and not associated with fractures in three (63 TPY) and five (105 TPY) analyses, respectively. Muscle mass can also be estimated using anthropometric prediction equations. The Lee equation includes height, weight, waist circumference, serum creatinine level and health behaviour factors.^39^ The Heymsfield equation is based on the triceps skinfold thickness and midarm circumference.^96^ A lower muscle mass derived from these two equations was positively and not associated with fractures in four (1381 TPY) and one (395 TPY) analyses, respectively. Using the creatine and creatinine‐derived parameters (D3‐creatine dilution test and estimated glomerular filtration rate [eGFR]), a lower parameter was associated with a higher, no and a lower fracture risk in 4 (170 TPY), 12 (507 TPY) and 2 (88 TPY) analyses, respectively.

### Muscle strength

Muscle strength was mostly assessed using the maximum isometric contraction of a specific muscle group. No analysis showed a negative association between muscle strength and fractures. A lower muscle strength was positively associated with incident fragility fractures in 53 (1.3 TPY) analyses and not associated in 57 (1.7 TPY) analyses. Hand grip strength (HGS) was associated with a higher and no fracture risk in 37 (1181 TPY) and 39 (1312 TPY) analyses, respectively. A lower triceps strength was associated with a higher and no fracture risk in two (29 TPY) and three (46 TPY) analyses, respectively. A lower quadriceps strength (QS) was associated with a higher and no fracture risk in 13 (131 TPY) and 15 (389 TPY) analyses, respectively. One study also analysed a lower arm and leg strength together and found a positive association (2 TPY) with fractures.

### Muscle function

Muscle function refers to tests that assess specific tasks, mobility and balance. As for muscle strength, none showed a negative association between muscle function's assessment and fractures. A lower muscle function was positively associated with incident fragility fracture in 63 (1901 TPY) analyses, not associated in 45 (972 TPY) analyses and negatively associated in 0 analyses. Gait speed (GS) refers to the usual walking speed over a distance of 4–6 m. A slower GS or loss of GS over time was associated with a higher and no fracture risk in 32 (1121 TPY) and 17 (391 TPY) analyses, respectively; it was positively associated with MOF in all the eight concerned studies (333 TPY).^31^, ^32^, ^41^, ^44^, ^54^, ^55^, ^58^ The different walking and chair rising tests were associated with a higher and no fracture risk in 19 (572 TPY) and 12 (299 TPY) analyses, respectively. They included five assessments: timed get up and go test (TGUG), change in TGUG, five‐time sit‐to‐stand test (5×STS), 𝚫 5×STS and squat/jump. Balance tests were associated with a higher and no fracture risk in 11 (184 TPY) and 10 (196 TPY) analyses, respectively. These included three different assessments: one‐leg standing test (OLST), narrow/tandem walk and single‐foot coordination. Multi‐item tests were associated with a higher and no fracture risk in one (24 TPY) and six (86 TPY) analyses, including three assessments: Short Physical Performance Battery (SPPB) test, sarcopenia screening questionnaire (SARC‐F) and a speed/reaction test.

## Discussion

In this scoping review, we investigated the association between 60 different muscle parameters with incident fractures risk in 322 separate analyses within 67 studies. Overall, low muscle mass was poorly/not associated with fracture risk, while low muscle strength and low muscle function were associated with higher risk of fracture. The results showed heterogeneity between the studies, in terms of studies' populations, measurement methods and statistical analysis. Our conclusion is a summary of the observed trends in this review and is not comparable to a meta‐analysis.

### Muscle mass, quantity and quality

Muscle mass, quantity and quality are objective and reproducible assessments of muscle health.^98^ The accuracy and the reliability of these assessments mostly depend on the technique used, for which the time available, the radiation dose, the costs and the patient involvement must also be considered. The gold standards are magnetic resonance imaging (MRI) and CT scan, but DXA and BIA remain the most widely used tools due to their easier accessibility.^99^, ^100^, ^101^ In this review, we did not find any studies using MRI. DXA and BIA were more studied as part of the diagnostic criteria of most sarcopenia definitions. The muscle quantity can be estimated from its volume using the muscle length and cross‐sectional area. As these two properties are also important components of muscle strength,^102^, ^103^ the hypothesis is that a low muscle quantity leads to weaker muscle (dynapaenia), which then lead to disbalance and falls.^104^ At the same time, we know that a tailored exercise programme reduces the risk of fall‐related fragility fractures.^105^ However, the relationship between low muscle mass and fractures has been repeatedly questioned.^12^, ^23^, ^45^ The results of our scoping review also suggest that a higher muscle mass, as assessed by different parameters, has little protective effect on the occurrence of fragility fractures. Indeed, seven analyses (within three studies) showed even opposite results with an increased risk of fragility fractures with higher muscle mass^44^, ^52^, ^66^: six (110 TPY) analyses for hip fractures and one (15 TPY) analysis for MOF. Interestingly, the analyses suggest that LM and ALM corrected for weight or BMI are mostly negatively or not associated with fragility fracture, whereas the same parameters corrected for height or height^2^ are mostly positively or not associated with fractures (*Figure* *3*).^40^, ^44^, ^45^, ^54^, ^55^, ^56^, ^57^, ^66^, ^67^ The use of LM indexes in fracture prediction models is complex because anthropometric measures are correlated with LM and are associated with fractures. The literature describes weight as a protective factor, height as a risk factor and BMI as having a U‐shaped association with fragility fractures.^106^ The stratification of LM analyses for body size or shape would enable a better estimation of its association with fragility fracture. Note that these considerations differ between the fragility fracture types and the sex (*Figure* *3* *A–E*). We also know that measures of LM include water, joints and ligaments^107^ and may not be specific enough of muscle mass.

Muscle density is a more recent concept. It was first used in CT scans by measuring the X‐ray absorption in the different muscle voxels (3D pixels) but is now also available in DXA.^66^ It is used as a proxy for intramuscular fat infiltration (as fat absorbs less X‐rays than bone or muscle) and has been associated with fragility fractures in this review.^37^, ^66^, ^73^ The bottleneck to more widespread use of CT scanning, including in larger studies, is the increased radiation dose and costs.

Muscle mass/quantity has also been investigated using biological tests, with promising results in fracture prediction. Blood creatine, a breakdown product of muscle, is associated with functional and clinical outcomes.^108^ Cystatin or its ratio showed a positive association in women with low eGFR and humerus fractures, but it showed conflicting results in men.^36^ Using the D3‐creatine dilution test, Cawthon et al. found a positive association between low eGFR and hip fractures and MOF.^33^ A review summarizes the necessary assumptions of the creatine dilution test, including individual variation (diet, age, activity level and disease state) that lead to underestimation or overestimation of the measurement.^108^ As a result, the clinical implementation of blood tests should be further investigated.

Newer methods are being developed such as ultrasound (e.g., with muscle thickness, cross‐sectional area, pennation angle and echogenicity)^109^ or image analysis (classification, segmentation, texture/pattern analysis and radiomics) using artificial intelligence (AI).^110^, ^111^ AI models could help us to extract the full information from the DXA scans (or other imaging modalities) and potentially measure new markers of muscle health. Pickhardt et al. analysed low‐dose CT scans using deep learning to predict lumbar muscle myosteatosis and cross‐sectional area.^112^ The prediction of hip fracture at 5 years was similar between their model (area under the curve [AUC] 0.709, 95% confidence interval [CI] 0.639–0.778) and the FRAX® (AUC 0.708, 95% CI 0.629–0.787).^112^ AI seems to be a suitable tool to analyse DXA body composition images and to search for unanticipated complex interactions between the available parameters.

The role of muscle mass in fragility fracture remains unclear. The assessment of muscle mass/quantity through the D3‐creatine dilution tests and muscle density assessment by DXA and CT imaging seem promising and could be object of further research. Furthermore, AI will undoubtedly influence musculoskeletal imaging and provide novel muscle mass assessments.

### Muscle strength

Muscle strength is highly correlated with muscle quantity (length and cross‐sectional area), but with greater variability,^102^ and is influenced by the conservation of peripheral and central neurological structures.^103^ Fifty per cent of the total body muscle mass lies in the lower body, while the upper body represents only 25%.^113^ Even if the quadriceps and psoas muscles make standing and walking possible, HGS has been shown to correlate with leg strength and is similarly predictive of low GS.^114^ From a clinical perspective, HGS is the most widely used test to assess muscle strength due to its low cost, accessibility, widespread use and reliability, whereas quadriceps testing is more complex and requires more equipment.^45^ This is probably the reason why fewer studies analysed QS. In this review, both lower HGS and lower QS were significantly associated with higher fracture risk in 37 and 13 (131 TPY) studies, respectively; 41 analyses showed no association between HGS and fracture risk and 15 (389 TPY) analyses between lower QS and fracture risk.

Muscle strength may be useful in predicting fracture risk using grip strength as a practical and reliable proxy of muscle strength.

### Muscle function

Muscle function is the most multifactorial determinant of muscle health. It correlates with both muscle mass and strength and is defined as the ability of the muscle to perform a certain task or movement. The assessment of muscle function, as for muscle strength, also depends on peripheral and central neurological structures. In addition, muscle function is closely linked to the brain (mostly through the cerebellum, motor, pre‐motor and supplementary motor cortex) when testing balance, coordination or complex tasks. The reasons for variation in measures of muscle function are similar to those for strength testing and are mainly analytical and/or methodological variations. Based on the observations of this review, GS shows a robust association with fracture risk, as all studies showed a significant association between slow GS and higher risk of MOF. The 5×STS was the second most commonly used muscle function test, with comparable results to QS. The 5×STS is a proxy of the thigh strength in addition to coordination ability. These observations emphasize the importance of assessing muscle function during a clinical consultation. Indeed, physicians are trained to assess the risk of falling (and therefore, to some extent, muscle function) by observing the patient walking around the examination room, sitting in the chair, changing clothes and so forth. For example, the chair stand tests (including 5×STS), the timed up and go test (TUGT), the SPPB and the tandem walk test have been validated to assess the mobility status and fall risk in older adults.^115^

Various muscle functional tests are available and provide an objective assessment of the patient muscle status, and they give an additional information on the patient's risk of fragility fracture. They include more variability than muscle strength or mass assessment but stay reliable overall. These tests were not designed to predict the fracture risk, but as they are associated with multiple medical conditions including neurological and musculoskeletal diseases, their association with fracture is also multifactorial.

### Clinical implications

In the field of sarcopenia, the association between muscle parameters and fragility fractures remains subject to debate. In the SDOC sarcopenia definition (2020), the authors argue against the use of muscle mass in further definitions because of insufficient evidence of its association with sarcopenia outcomes (including fractures) and the cost of DXA.^45^ Our scoping review similarly suggests that low muscle mass, as currently defined, is not robustly associated with fragility fractures and that an adjustment or stratification for body size is necessary. As we analysed each muscle health component separately and did not assess the other sarcopenia endpoints, our study does not allow us to directly challenge the composite definitions of sarcopenia. On the other hand, the observed association of GS and HGS with fragility fractures supports their use in the diagnostic workflow of current sarcopenia definitions. These muscle parameters provide objective measures of the muscle health and insights on its association with fragility fractures. Ideally, a test or score would be developed to specifically identify the fracture risk associated with sarcopenia, at best independently from the risk of fall.

In the field of osteoporosis, the relationship between bone and muscle has been studied from various angles. Falls are important risk factors for fracture occurrence. They often, but not always, precede the fracture.^9^ In the causal hypothesis linking muscle mass to fragility fractures, falls are more likely to be a mediator in the equation, involving both dependent and independent pathways, rather than just an intermediate factor. In this scoping review, only few studies demonstrated that the relation between muscle mass,^33^, ^37^, ^55^, ^57^ strength^69^ and function^31^, ^32^, ^34^, ^55^, ^64^, ^69^ with incident fracture was positive and independent from falls. At the cellular level, a cross‐talk between muscle and bone has been discussed in studies about osteo‐sarcopenia.^13^ At the organ level, the bone mechanostat hypothesis explains that the properties of load‐bearing bones are primarily influenced by their functions, rather than the influence of load and gravitational forces.^116^ Our study could support this hypothesis considering that muscle function and strength have an additive discriminative value in fragility fractures prediction models, assuming that bone properties are related in the same way. However, muscle mass and quantity, as it currently stands, do not appear to have an independent effect on fracture susceptibility. Heymsfield et al. insisted on the importance of muscle ‘form’ (size and shape) and not only muscle function in the pathophysiology of adverse events (cf. OFF hypothesis: Outcome follow function, follow form), based on the axiom that without the physical form of the muscle, there would be no function.^117^ The overall lack of association between muscle mass/quantity and fractures that we highlight in this review does not discredit its importance in the pathophysiology of osteoporosis and sarcopenia. Further research is needed on muscle mass, quantity and quality in the prediction of fracture risk, including a judicious use of anthropometric measures. The D3‐creatine dilution test and the CT‐scan measures showed promising results, while LM, its indexes and the new statistical approaches using AI need to be further investigated.

Muscle health parameters are important in the prevention and diagnostic of sarcopenia and in the assessment of osteoporotic patients. This scoping review highlights the benefits and the gaps of muscle health tests in clinical setting and in community‐dwelling older adults.

### Strengths and limitations

This study has some limitations. First, a common limitation to scoping reviews is the publication bias. Positive studies are more likely to be published, whereas negative studies may be discontinued. However, most of the results analysed are inconclusive (no association) and some are even negative and contra‐intuitive (e.g., the positive association between muscle mass and fragility fracture risk), suggesting that the data observed and discussed here are undistorted. Second, the overall quality and risk of bias of the included studies were not systematically assessed. However, this is not a requirement for conducting a scoping review. As shown in *Tables* *1* and *2*, the majority of the included studies have large sample sizes and long follow‐up periods and come from recognized and well‐conducted national or international cohorts. Finally, although not related to the scoping review itself, the included studies have some limitations that weaken their interpretation, such as the consideration of non‐MOF fractures as fragility fractures (*Figures* *S3f* *–* *S5f*); the lack of a clear fragility fracture definition^30^, ^34^, ^41^, ^69^, ^71^, ^78^, ^83^, ^88^, ^90^; and the lack of systematic radiographic assessment for fracture detection, as some incident fractures were only collected based on questionnaires and general practitioners.

To the best of our knowledge, this is the first review, based on a systematic search, that thoroughly reviews studies that investigated the association of incident fracture risk with muscle mass/quantity/quality, strength and/or functional parameters. The rigorous systematic search, under the supervision of medical library experts, adds value to the current study. The inclusion of only prospective studies is a major strength, as prospective studies have a temporal framework to assess causality (outcome occurring after exposure), which positions them as strong scientific evidence. In addition, most of the analyses were performed with the muscle parameter as a continuous variable, assuming that the risk is proportional to the parameter in question. Some studies had previously categorized the variables using percentiles or a specific value (cf. *Figures* *3*, *4*, *5*), which lost statistical information but made it easier to use in clinical practice. Furthermore, following the PRISMA checklist for reporting (cf. supporting information) and the JBI methodology for writing improves the transparency, reproducibility and, ultimately, the overall quality of this review. Moreover, we visualize the trend of associations between muscle parameters and fracture risk using adapted forest plots. Finally, our review highlights muscle parameters that could be further analysed in a meta‐analysis.

## Conclusions

This scoping review gives a broad overview of the gaps and evidences in the relationship between muscle parameters and fragility fractures. Poorer muscle function followed by lower muscle strength were the parameters mostly related to a higher risk of incident fragility fractures. For daily clinical practice, this review suggests that measures of HGS and GS are the most useful methods to assess muscle‐dependent fracture risk. This supports their use in the evaluation of sarcopenia. This review also confirms that muscle mass, as currently defined, is a poor independent predictor of fragility fracture. For future research and development of fragility fracture prediction models, it will be necessary to determine whether muscle‐associated fracture risk is fully independent from other risk factors. In addition, further investigation of DXA images, including body composition, using AI methods may reveal new complex interactions between muscle tissue and fragility fractures.

## Conflict of interest statement

Colin Vendrami, Enisa Shevroja, Guillaume Gatineau, Jolanda Elmers, Elena Gonzalez Rodriguez, Jean‐Yves Reginster, Nicholas C. Harvey, Olivier Lamy and Didier Hans declare that they have no conflict of interest related to this manuscript.

## Supporting information


**Data S1.** Search equations syntaxes – Part I.
**Data S2.** Search equations syntaxes – Part II.
**Table S1.** Preferred Reporting Items for Systematic Reviews and Meta‐Analysis extension for Scoping Reviews (PRISMA‐ScR) checklist – Part I.
**Table S1.** Preferred Reporting Items for Systematic Reviews and Meta‐Analysis extension for Scoping Reviews (PRISMA‐ScR) checklist – Part II.
**Table S2.** Summary of the main analysis for each muscle assessment and each fracture types including the gaps.
**Figure S3f.** Muscle mass parameters and risk of incident fragility fractures: All fragility fractures.
**Figure S4f.** Muscle strength parameters and risk of incident fragility fractures: All fragility fractures.
**Figure S5f.** Muscle function parameters and risk of incident fragility fractures: All fragility fractures.

## References

[1] Bonjour J‐P , Couper M , Dr S , Dutta , Fracchia G , Gundert‐Remy U , et al. Guidelines for preclinical evaluation and clinical trials in osteoporosis. Geneva: World Health Organization; 1998. 74 p.

[2] Kanis JA , Norton N , Harvey NC , Jacobson T , Johansson H , Lorentzon M , et al. SCOPE 2021: a new scorecard for osteoporosis in Europe. Arch Osteoporos 2021;16:82. 34080059 10.1007/s11657-020-00871-9 PMC8172408

[3] Lippuner K , Grifone S , Schwenkglenks M , Schwab P , Popp AW , Senn C , et al. Comparative trends in hospitalizations for osteoporotic fractures and other frequent diseases between 2000 and 2008. Osteoporos Int J Establ Result Coop Eur Found Osteoporos Natl Osteoporos Found USA 2012;23:829–839. 10.1007/s00198-011-1660-8 21625882

[4] United Nations, Department of Economic and Social Affairs, Population Division . World population prospects Highlights, 2019 revision Highlights, 2019 revision. 2019.

[5] Hernlund E , Svedbom A , Ivergård M , Compston J , Cooper C , Stenmark J , et al. Osteoporosis in the European Union: medical management, epidemiology and economic burden: a report prepared in collaboration with the International Osteoporosis Foundation (IOF) and the European Federation of Pharmaceutical Industry Associations (EFPIA). Arch Osteoporos 2013;8:10.1007/s11657-013-0136-1 PMC3880487 24113837

[6] Bonjour P , Dawson‐Hughes B , De Laet C , Johansson H , Johnell O , Melton J , et al. WHO Scientific Group on the assessment of osteoporosis at primary health care level. Brussels, Belgium: WHO; 2004. p 17 Available from: https://www.who.int/chp/topics/Osteoporosis.pdf

[7] Siris ES , Chen YT , Abbott TA , Barrett‐Connor E , Miller PD , Wehren LE , et al. Bone mineral density thresholds for pharmacological intervention to prevent fractures. Arch Intern Med 2004;164:1108–1112. 15159268 10.1001/archinte.164.10.1108

[8] Kanis JA , Johansson H , Harvey NC , McCloskey EV . A brief history of FRAX. Arch Osteoporos 2018;13:118. 30382424 10.1007/s11657-018-0510-0 PMC6290984

[9] Kanis JA , Johansson H , Harvey NC , Lorentzon M , Liu E , Vandenput L , et al. Adjusting conventional FRAX estimates of fracture probability according to the number of prior falls in the preceding year. Osteoporos Int J Establ Result Coop Eur Found Osteoporos Natl Osteoporos Found USA 2023;34:479–487. 10.1007/s00198-022-06633-2 36562788

[10] Lexell J , Taylor CC , Sjöström M . What is the cause of the ageing atrophy? J Neurol Sci 1988;84:275–294. 3379447 10.1016/0022-510x(88)90132-3

[11] Rosenberg IH . Sarcopenia: origins and clinical relevance. J Nutr 1997;127:990S–991S. 9164280 10.1093/jn/127.5.990S

[12] Cruz‐Jentoft AJ , Bahat G , Bauer J , Boirie Y , Bruyère O , Cederholm T , et al. Sarcopenia: revised European consensus on definition and diagnosis. Age Ageing 2019;48:16–31. 30312372 10.1093/ageing/afy169 PMC6322506

[13] Hirschfeld HP , Kinsella R , Duque G . Osteosarcopenia: where bone, muscle, and fat collide. Osteoporos Int 2017;28:2781–2790. 28733716 10.1007/s00198-017-4151-8

[14] Merchant RA , Chen MZ , Wong BLL , Ng SE , Shirooka H , Lim JY , et al. Relationship between fear of falling, fear‐related activity restriction, frailty, and sarcopenia. J Am Geriatr Soc 2020;68:2602–2608. 32804411 10.1111/jgs.16719

[15] Beaudart C , Reginster JY , Amuthavalli Thiyagarajan J , Bautmans I , Bauer J , Burlet N , et al. Measuring health‐related quality of life in sarcopenia: summary of the SarQoL psychometric properties. Aging Clin Exp Res 2023;35:1581–1593. 37219755 10.1007/s40520-023-02438-3 PMC10363087

[16] Bahat G , Bozkurt ME , Ozkok S , Kilic C , Karan MA . The longitudinal associations of sarcopenia definitions with functional deterioration: a comparative study. Aging Clin Exp Res 2023;35:2089–2099. 37486546 10.1007/s40520-023-02498-5

[17] Yeung SSY , Reijnierse EM , Pham VK , Trappenburg MC , Lim WK , Meskers CGM , et al. Sarcopenia and its association with falls and fractures in older adults: a systematic review and meta‐analysis. J Cachexia Sarcopenia Muscle 2019;10:485–500. 30993881 10.1002/jcsm.12411 PMC6596401

[18] Bertschi D , Kiss CM , Beerli N , Mauthner O , Kressig RW . Impact of sarcopenia on daily functioning: a cross‐sectional study among older inpatients. Aging Clin Exp Res 2022;34:2041–2046. 35794312 10.1007/s40520-022-02175-z PMC9464162

[19] Beaudart C , Zaaria M , Pasleau F , Reginster JY , Bruyère O . Health outcomes of sarcopenia: a systematic review and meta‐analysis. Wright JM, editor. PLoS ONE 2017;12:e0169548. 28095426 10.1371/journal.pone.0169548 PMC5240970

[20] Mijnarends DM , Luiking YC , Halfens RJG , Evers SMAA , Lenaerts ELA , Verlaan S , et al. Muscle, health and costs: a glance at their relationship. J Nutr Health Aging 2018;22:766–773. 30080217 10.1007/s12603-018-1058-9 PMC6061527

[21] Sousa AS , Guerra RS , Fonseca I , Pichel F , Ferreira S , Amaral TF . Financial impact of sarcopenia on hospitalization costs. Eur J Clin Nutr 2016;70:1046–1051. 27167668 10.1038/ejcn.2016.73

[22] Bruyère O , Beaudart C , Ethgen O , Reginster JY , Locquet M . The health economics burden of sarcopenia: a systematic review. Maturitas 2019;119:61–69. 30502752 10.1016/j.maturitas.2018.11.003

[23] Stuck AK , Basile G , Freystaetter G , de Godoi Rezende Costa Molino C , Lang W , Bischoff‐Ferrari HA . Predictive validity of current sarcopenia definitions (EWGSOP2, SDOC, and AWGS2) for clinical outcomes: a scoping review. J Cachexia Sarcopenia Muscle 2022;14:jcsm.13161. 10.1002/jcsm.13161 PMC9891988 36564353

[24] Harvey NC , Orwoll E , Kwok T , Karlsson MK , Rosengren BE , Ribom E , et al. Sarcopenia definitions as predictors of fracture risk independent of frax ®, falls, and bmd in the Osteoporotic Fractures in Men (mros) Study: a meta‐analysis. J Bone Miner Res 2021;36:1235–1244. 33831257 10.1002/jbmr.4293 PMC7611727

[25] Peters MDJ , Godfrey CM , McInerney P , Munn Z , Tricco A , Khalil H . Chapter 11: scoping reviews (2020 version). In Aromataris E , Munn Z , eds. JBI manual for evidence synthesis. JBI; 2020. Available from: https://synthesismanual.jbi.global

[26] Tricco AC , Lillie E , Zarin W , O'Brien KK , Colquhoun H , Levac D , et al. PRISMA extension for Scoping Reviews (PRISMA‐ScR): checklist and explanation. Ann Intern Med 2018;169:467–473. 30178033 10.7326/M18-0850

[27] Ouzzani M , Hammady H , Fedorowicz Z , Elmagarmid A . Rayyan—a web and mobile app for systematic reviews. Syst Rev 2016;5:210. 27919275 10.1186/s13643-016-0384-4 PMC5139140

[28] Cawthon PM , Visser M , Arai H , Ávila‐Funes JA , Barazzoni R , Bhasin S , et al. Defining terms commonly used in sarcopenia research: a glossary proposed by the Global Leadership in Sarcopenia (GLIS) Steering Committee. Eur Geriatr Med 2022;13:1239–1244. 36445639 10.1007/s41999-022-00706-5 PMC9722886

[29] Justice AC , Covinsky KE , Berlin JA . Assessing the generalizability of prognostic information. Ann Intern Med 1999;130:515–524. 10075620 10.7326/0003-4819-130-6-199903160-00016

[30] Yamada M , Kimura Y , Ishiyama D , Otobe Y , Suzuki M , Koyama S , et al. Combined effect of lower muscle quality and quantity on incident falls and fall‐related fractures in community‐dwelling older adults: a 3‐year follow‐up study. Bone 2022;162:116474. 35752409 10.1016/j.bone.2022.116474

[31] Harris RJ , Parimi N , Cawthon PM , Strotmeyer ES , Boudreau RM , Brach JS , et al. Associations of components of sarcopenia with risk of fracture in the Osteoporotic Fractures in Men (MrOS) study. Osteoporos Int 2022; 10.1007/s00198-022-06390-2 PMC10011872 35380213

[32] Fujita Y , Iki M , Yura A , Harano A , Kouda K , Tamaki J , et al. Combined results of three physical performance tests predict incident fracture independently of aBMD in community‐dwelling elderly Japanese men: Fujiwara‐kyo Osteoporosis Risk in Men (FORMEN) Cohort Study. Bone 2022;154:116240. 34678493 10.1016/j.bone.2021.116240

[33] Cawthon PM , Peters KE , Cummings SR , Orwoll ES , Hoffman AR , Ensrud KE , et al. Association between muscle mass determined by D3‐creatine dilution and incident fractures in a prospective cohort study of older men. J Bone Miner Res 2022;jbmr.4505. 10.1002/jbmr.4505 PMC9283198 35253257

[34] Zhong BX , Zhong HL , Zhou GQ , Xu WQ , Lu Y , Zhao Q . Physical performance and risk of hip fracture in community‐dwelling elderly people in China: a 4‐year longitudinal cohort study. Maturitas 2021;146:26–33. 33722361 10.1016/j.maturitas.2021.01.003

[35] Westbury LD , Syddall HE , Fuggle NR , Dennison EM , Harvey NC , Cauley JA , et al. Relationships between level and change in sarcopenia and other body composition components and adverse health outcomes: findings from the Health, Aging, and Body Composition Study. Calcif Tissue Int 2021;108:302–313. 33191483 10.1007/s00223-020-00775-3 PMC7881954

[36] Nordvåg SK , Solbu MD , Melsom T , Nissen FI , Andreasen C , Borgen TT , et al. Estimated glomerular filtration rate (eGFR) based on cystatin C was associated with increased risk of hip and proximal humerus fractures in women and decreased risk of hip fracture in men, whereas eGFR based on creatinine was not associated with fracture risk in both sexes: the Tromsø Study. Bone 2021;148:115960. 33864977 10.1016/j.bone.2021.115960

[37] Harvey NC . Greater PQCT calf muscle density is associated with lower hip fracture risk, independent of FRAX, falls and BMD: a meta‐analysis in the Osteoporotic Fractures in Men (MrOS) Study. [abstract] [Internet]. Poster abstract presented at: World Congress on Osteoporosis, Osteoarthritis and Musculoskeletal Diseases (WCO‐IOF‐ESCEO 2021). 2021. London. Available from: https://link.springer.com/10.1007/s00198‐021‐06125‐9

[38] McGrath R , Blackwell TL , Ensrud KE , Vincent BM , Cawthon PM . The associations of handgrip strength and leg extension power asymmetry on incident recurrent falls and fractures in older men. J Gerontol A Biol Sci Med Sci 2021;76:e221–e227. 33978154 10.1093/gerona/glab133 PMC8499308

[39] Hong C , Choi S , Park M , Park SM , Lee G . Body composition and osteoporotic fracture using anthropometric prediction equations to assess muscle and fat masses. J Cachexia Sarcopenia Muscle 2021;12:2247–2258. 34706399 10.1002/jcsm.12850 PMC8718033

[40] Harvey NC , Kanis JA , Liu E , Cooper C , Lorentzon M , Bea JW , et al. Predictive value of DXA appendicular lean mass for incident fractures, falls, and mortality, independent of prior falls, FRAX, and BMD: findings from the Women's Health Initiative (WHI). J Bone Miner Res 2021;36:654–661. 33450071 10.1002/jbmr.4239 PMC7610603

[41] Alajlouni D , Tran T , Bliuc D , Blank RD , Cawthon PM , Orwoll ES , et al. Muscle strength and physical performance improve fracture risk prediction beyond Garvan and FRAX: the Osteoporotic Fractures in Men (MrOS) Study. J Bone Miner Res 2021;37:411–419. 34842309 10.1002/jbmr.4483 PMC8940659

[42] Søgaard AJ , Magnus JH , Bjørnerem Å , Holvik K , Ranhoff AH , Emaus N , et al. Grip strength in men and women aged 50–79 years is associated with non‐vertebral osteoporotic fracture during 15 years follow‐up: the Tromsø Study 1994–1995. Osteoporos Int 2020;31:131–140. 31650188 10.1007/s00198-019-05191-4

[43] Leslie WD , Schousboe JT , Morin SN , Martineau P , Lix LM , Johansson H , et al. Loss in DXA‐estimated total body lean mass but not fat mass predicts incident major osteoporotic fracture and hip fracture independently from FRAX: a registry‐based cohort study. Arch Osteoporos 2020;15:96. 32588147 10.1007/s11657-020-00773-w PMC7115892

[44] Lam FMH , Su Y , Lu ZH , Yu R , Leung JCS , Kwok TCY . Cumulative and incremental value of sarcopenia components on predicting adverse outcomes. J Am Med Dir Assoc 2020;21:1481–1489.e3. 32768375 10.1016/j.jamda.2020.05.056 PMC8831392

[45] Cawthon PM , Manini T , Patel SM , Newman A , Travison T , Kiel DP , et al. Putative cut‐points in sarcopenia components and incident adverse health outcomes: an SDOC analysis. J Am Geriatr Soc 2020;68:1429–1437. 32633824 10.1111/jgs.16517 PMC7508260

[46] Alajlouni D , Bliuc D , Tran T , Eisman JA , Nguyen TV , Center JR . Decline in muscle strength and performance predicts fracture risk in elderly women and men. J Clin Endocrinol Metab 2020;105:dgaa414. 32639571 10.1210/clinem/dgaa414

[47] Scott D , Seibel M , Cumming R , Naganathan V , Blyth F , Le Couteur DG , et al. Does combined osteopenia/osteoporosis and sarcopenia confer greater risk of falls and fracture than either condition alone in older men? The Concord Health and Ageing in Men Project. J Gerontol A Biol Sci Med Sci 2019;74:827–834. 30032209 10.1093/gerona/gly162

[48] Kamiya K , Kajita E , Tachiki T , Ikehara S , Kouda K , Sato Y , et al. Association between hand‐grip strength and site‐specific risks of major osteoporotic fracture: results from the Japanese Population‐based Osteoporosis Cohort Study. Maturitas 2019;130:13–20. 31706431 10.1016/j.maturitas.2019.09.008

[49] Cronholm F , Rosengren BE , Nilsson JÅ , Ohlsson C , Mellström D , Ribom E , et al. The fracture predictive ability of a musculoskeletal composite score in old men—data from the MrOs Sweden study. BMC Geriatr 2019;19:90. 30902044 10.1186/s12877-019-1106-2 PMC6431016

[50] Wright NC , Hooker ER , Nielson CM , Ensrud KE , Harrison SL , Orwoll ES , et al. The epidemiology of wrist fractures in older men: the Osteoporotic Fractures in Men (MrOS) study. Osteoporos Int J Establ Result Coop Eur Found Osteoporos Natl Osteoporos Found USA 2018;29:859–870. 10.1007/s00198-017-4349-9 PMC5939930 29344692

[51] Schaap LA , van Schoor NM , Lips P , Visser M . Associations of sarcopenia definitions, and their components, with the incidence of recurrent falling and fractures: the Longitudinal Aging Study Amsterdam. J Gerontol Ser A 2018;73:1199–1204. 10.1093/gerona/glx245 29300839

[52] McLean RR , Kiel DP , Berry SD , Broe KE , Zhang X , Cupples LA , et al. Lower lean mass measured by dual‐energy X‐ray absorptiometry (DXA) is not associated with increased risk of hip fracture in women: the Framingham Osteoporosis Study. Calcif Tissue Int 2018;103:16–23. 29305636 10.1007/s00223-017-0384-y PMC6013345

[53] Kim JH , Hong AR , Choi HJ , Ku EJ , Lee JH , Cho NH , et al. Defining sarcopenia in terms of skeletal health. Arch Osteoporos 2018;13:100. 30238222 10.1007/s11657-018-0511-z

[54] Harvey NC , Odén A , Orwoll E , Lapidus J , Kwok T , Karlsson MK , et al. Measures of physical performance and muscle strength as predictors of fracture risk independent of FRAX, falls, and aBMD: a meta‐analysis of the Osteoporotic Fractures in Men (MrOS) Study. J Bone Miner Res 2018;33:2150–2157. 30011086 10.1002/jbmr.3556 PMC6272117

[55] Buehring B , Hansen KE , Lewis BL , Cummings SR , Lane NE , Binkley N , et al. Dysmobility syndrome independently increases fracture risk in the Osteoporotic Fractures in Men (MrOS) prospective cohort study. J Bone Miner Res 2018;33:1622–1629. 29701911 10.1002/jbmr.3455 PMC6469960

[56] Zaslavsky O , Li W , Going S , Datta M , Snetselaar L , Zelber‐Sagi S . Association between body composition and hip fractures in older women with physical frailty: adiposity and hip fracture in frailty. Geriatr Gerontol Int 2017;17:898–904. 27164296 10.1111/ggi.12798 PMC5104679

[57] Sornay‐Rendu E , Duboeuf F , Boutroy S , Chapurlat R . Muscle mass is associated with incident fracture in postmenopausal women: the OFELY study. Bone 2017;94:108–113. 27989649 10.1016/j.bone.2016.10.024

[58] Lundin H , Sääf M , Strender LE , Nyren S , Johansson SE , Salminen H . Gait speed and one‐leg standing time each add to the predictive ability of FRAX. Osteoporos Int 2017;28:179–187. 27844133 10.1007/s00198-016-3818-x PMC5206249

[59] Lee EY , Lee SJ , Kim KM , Seo DH , Lee SW , Choi HS , et al. Lower jump power rather than muscle mass itself is associated with vertebral fracture in community‐dwelling elderly Korean women. Calcif Tissue Int 2017;100:585–594. 28275826 10.1007/s00223-017-0239-6

[60] Harris R , Chang Y , Beavers K , Laddu‐Patel D , Bea J , Johnson K , et al. Risk of fracture in women with sarcopenia, low bone mass, or both. J Am Geriatr Soc 2017;65:2673–2678. 28960230 10.1111/jgs.15050 PMC5729083

[61] Balogun S , Winzenberg T , Wills K , Scott D , Jones G , Aitken D , et al. Prospective associations of low muscle mass and function with 10‐year falls risk, incident fracture and mortality in community‐dwelling older adults. J Nutr Health Aging 2017;21:843–848.28717816 10.1007/s12603-016-0843-6

[62] Pham HM , Nguyen ND , Center JR , Eisman JA , Nguyen TV . Contribution of quadriceps weakness to fragility fracture: a prospective study: Quadriceps weakness and fracture risk. J Bone Miner Res 2016;31:208–214. 26174768 10.1002/jbmr.2594

[63] Hars M , Biver E , Chevalley T , Herrmann F , Rizzoli R , Ferrari S , et al. Low lean mass predicts incident fractures independently from FRAX: a prospective cohort study of recent retirees. J Bone Miner Res 2016;31:2048–2056. 27253633 10.1002/jbmr.2878

[64] Barbour KE , Lui LY , McCulloch CE , Ensrud KE , Cawthon PM , Yaffe K , et al. Trajectories of lower extremity physical performance: effects on fractures and mortality in older women. J Gerontol A Biol Sci Med Sci 2016;71:1609–1615. 27084313 10.1093/gerona/glw071 PMC5106858

[65] Wihlborg A , Englund M , Åkesson K , Gerdhem P . Fracture predictive ability of physical performance tests and history of falls in elderly women: a 10‐year prospective study. Osteoporos Int 2015;26:2101–2109. 25832178 10.1007/s00198-015-3106-1

[66] Malkov S , Cawthon PM , Peters KW , Cauley JA , Murphy RA , Visser M , et al. Hip fractures risk in older men and women associated with DXA‐derived measures of thigh subcutaneous fat thickness, cross‐sectional muscle area, and muscle density. J Bone Miner Res Off J Am Soc Bone Miner Res 2015;30:1414–1421. 10.1002/jbmr.2469 PMC5111546 25644748

[67] Cawthon PM , Blackwell TL , Cauley J , Kado DM , Barrett‐Connor E , Lee CG , et al. An evaluation of the usefulness of consensus definitions of sarcopenia in older men: results from the observational Osteoporotic Fractures in Men (MrOS) cohort study. J Am Geriatr Soc 2015;63:2247–2259. 26502831 10.1111/jgs.13788 PMC4739621

[68] Yu R , Leung J , Woo J . Incremental predictive value of sarcopenia for incident fracture in an elderly Chinese cohort: results from the Osteoporotic Fractures in Men (MrOs) Study. J Am Med Dir Assoc 2014;15:551–558. 24703927 10.1016/j.jamda.2014.02.005

[69] Ryg J , Vestergaard S , Lindholm Eriksen M , Andersen‐Ranberg K , Masud T . Hip fractures are predicted by functional tests in a large European ageing study. Eur Geriatr Med 2013;4:S60.

[70] Rouzi AA , Al‐Sibiani SA , Al‐Senani NS , Radaddi RM , Ardawi MSM . Independent predictors of all osteoporosis‐related fractures among healthy Saudi postmenopausal women: the CEOR Study. Bone 2012;50:713–722. 22178778 10.1016/j.bone.2011.11.024

[71] Edwards M , Jameson K , Gregson C , Harvey N , Sayer AA , Dennison E , et al. Muscle size, strength and physical performance as predictors of falls and fractures in the Hertfordshire Cohort Study. Osteoporos Int 2012;23:S555. 10.1002/jbmr.1972 PMC3805465 23633238

[72] Cheung CL , Tan KCB , Bow CH , Soong CSS , Loong CHN , Kung AWC . Low handgrip strength is a predictor of osteoporotic fractures: cross‐sectional and prospective evidence from the Hong Kong Osteoporosis Study. Age (Dordr) 2012;34:1239–1248. 21853264 10.1007/s11357-011-9297-2 PMC3448988

[73] Lang T , Cauley JA , Tylavsky F , Bauer D , Cummings S , Harris TB , et al. Computed tomographic measurements of thigh muscle cross‐sectional area and attenuation coefficient predict hip fracture: the Health, Aging, and Body Composition Study. J Bone Miner Res Off J Am Soc Bone Miner Res 2010;25:513–519. 10.1359/jbmr.090807 PMC3153392 20422623

[74] Sirola J , Rikkonen T , Tuppurainen M , Jurvelin JS , Alhava E , Kröger H . Grip strength may facilitate fracture prediction in perimenopausal women with normal BMD: a 15‐year population‐based study. Calcif Tissue Int 2008;83:93–100. 18641912 10.1007/s00223-008-9155-0

[75] Kärkkäinen M , Rikkonen T , Kröger H , Sirola J , Tuppurainen M , Salovaara K , et al. Association between functional capacity tests and fractures: an eight‐year prospective population‐based cohort study. Osteoporos Int 2008;19:1203–1210. 18236100 10.1007/s00198-008-0561-y

[76] Finigan J , Greenfield DM , Blumsohn A , Hannon RA , Peel NF , Jiang G , et al. Risk factors for vertebral and nonvertebral fracture over 10 years: a population‐based study in women. J Bone Miner Res 2008;23:75–85. 17784843 10.1359/jbmr.070814

[77] Cawthon PM , Fullman RL , Marshall L , Mackey DC , Fink HA , Cauley JA , et al. Physical performance and risk of hip fractures in older men. J Bone Miner Res 2008;23:1037–1044. 18302496 10.1359/JBMR.080227 PMC2679379

[78] Nguyen ND , Eisman JA , Center JR , Nguyen TV . Risk factors for fracture in nonosteoporotic men and women. J Clin Endocrinol Metab 2007;92:955–962. 17164302 10.1210/jc.2006-1476

[79] Sipilä S , Heikkinen E , Cheng S , Suominen H , Saari P , Kovanen V , et al. Endogenous hormones, muscle strength, and risk of fall‐related fractures in older women. J Gerontol Ser A 2006;61:92–96. 10.1093/gerona/61.1.92 16456199

[80] Shigematsu R , Rantanen T , Saari P , Sakari‐Rantala R , Kauppinen M , Sipilä S , et al. Motor speed and lower extremity strength as predictors of fall‐related bone fractures in elderly individuals. Aging Clin Exp Res 2006;18:320–324. 17063067 10.1007/BF03324666

[81] Samelson EJ , Hannan MT , Zhang Y , Genant HK , Felson DT , Kiel DP . Incidence and risk factors for vertebral fracture in women and men: 25‐year follow‐up results from the population‐based Framingham study. J Bone Miner Res 2006;21:1207–1214. 16869718 10.1359/jbmr.060513

[82] Pluijm SMF , Smit JH , Tromp E a M , Stel VS , Deeg DJH , Bouter LM , et al. A risk profile for identifying community‐dwelling elderly with a high risk of recurrent falling: results of a 3‐year prospective study. Osteoporos Int 2006;17:417–425. 16416256 10.1007/s00198-005-0002-0

[83] Robbins JA , Schott AM , Garnero P , Delmas PD , Hans D , Meunier PJ . Risk factors for hip fracture in women with high BMD: EPIDOS study. Osteoporos Int 2005;16:149–154. 15185066 10.1007/s00198-004-1661-y

[84] Nguyen ND , Pongchaiyakul C , Center JR , Eisman JA , Nguyen TV . Identification of high‐risk individuals for hip fracture: a 14‐year prospective study. J Bone Miner Res 2005;20:1921–1928. 16234964 10.1359/JBMR.050520

[85] Dixon WG , Lunt M , Pye SR , Reeve J , Felsenberg D , Silman AJ , et al. Low grip strength is associated with bone mineral density and vertebral fracture in women. Rheumatology 2005;44:642–646. 15728415 10.1093/rheumatology/keh569

[86] Albrand G , Munoz F , Sornay‐Rendu E , DuBoeuf F , Delmas PD . Independent predictors of all osteoporosis‐related fractures in healthy postmenopausal women: the OFELY Study. Bone 2003;32:78–85. 12584039 10.1016/s8756-3282(02)00919-5

[87] Lee SH , Dargent‐Molina P , Bréart G . Risk factors for fractures of the proximal humerus: results from the EPIDOS prospective study. J Bone Miner Res 2002;17:817–825. 12009012 10.1359/jbmr.2002.17.5.817

[88] Dargent‐Molina P , Schott AM , Hans D , Favier F , Grandjean H , Baudoin C , et al. Separate and combined value of bone mass and gait speed measurements in screening for hip fracture risk: results from the EPIDOS study. Osteoporos Int 1999;9:188–192. 10367048 10.1007/s001980050134

[89] Nguyen TV , Eisman JA , Kelly PJ , Sambrook PN . Risk factors for osteoporotic fractures in elderly men. Am J Epidemiol 1996;144:255–263. 8686694 10.1093/oxfordjournals.aje.a008920

[90] Dargent‐Molina P , Favier F , Grandjean H , Baudoin C , Schott A , Hausherr E , et al. Fall‐related factors and risk of hip fracture: the EPIDOS prospective study. Lancet 1996;348:145–149. 8684153 10.1016/s0140-6736(96)01440-7

[91] Cummings SR , Nevitt MC , Browner WS , Stone K , Fox KM , Ensrud KE , et al. Risk factors for hip fracture in white women. N Engl J Med 1995;332:767–773. 7862179 10.1056/NEJM199503233321202

[92] Nguyen T , Sambrook P , Kelly P , Jones G , Lord S , Freund J , et al. Prediction of osteoporotic fractures by postural instability and bone density. BMJ 1993;307:1111–1115. 8251809 10.1136/bmj.307.6912.1111 PMC1679116

[93] Nevitt MC , Cummings SR , Group S of OFR . Type of fall and risk of hip and wrist fractures: the study of osteoporotic fractures. J Am Geriatr Soc 1993;41:1226–1234. 8227898 10.1111/j.1532-5415.1993.tb07307.x

[94] Kelsey JL , Browner WS , Seeley DG , Nevitt MC , Cummings SR . Risk factors for fractures of the distal forearm and proximal humerus. Am J Epidemiol 1992;135:477–489. 1570814 10.1093/oxfordjournals.aje.a116314

[95] Wickham CA , Walsh K , Cooper C , Barker DJ , Margetts BM , Morris J , et al. Dietary calcium, physical activity, and risk of hip fracture: a prospective study. BMJ 1989;299:889–892. 2510879 10.1136/bmj.299.6704.889 PMC1837771

[96] Farmer ME , Harris T , Madans JH , Wallace RB , Cornoni‐Huntley J , White LR . Anthropometric indicators and hip fracture: the NHANES I epidemiologic follow‐up study. J Am Geriatr Soc 1989;37:9–16. 2909610 10.1111/j.1532-5415.1989.tb01562.x

[97] Peters MDJ , Godfrey CM , Khalil H , McInerney P , Parker D , Soares CB . Guidance for conducting systematic scoping reviews. JBI Evid Implement 2015;13:141–146. 10.1097/XEB.0000000000000050 26134548

[98] Hangartner TN , Warner S , Braillon P , Jankowski L , Shepherd J . The official positions of the International Society for Clinical Densitometry: acquisition of dual‐energy X‐ray absorptiometry body composition and considerations regarding analysis and repeatability of measures. J Clin Densitom 2013;16:520–536. 24183641 10.1016/j.jocd.2013.08.007

[99] Ackermans LLGC , Rabou J , Basrai M , Schweinlin A , Bischoff SC , Cussenot O , et al. Screening, diagnosis and monitoring of sarcopenia: when to use which tool? Clin Nutr ESPEN 2022;48:36–44. 35331514 10.1016/j.clnesp.2022.01.027

[100] Shepherd J , Ng B , Sommer M , Heymsfield SB . Body composition by DXA. Bone 2017;104:101–105. 28625918 10.1016/j.bone.2017.06.010 PMC5659281

[101] McCarthy C , Tinsley GM , Bosy‐Westphal A , Müller MJ , Shepherd J , Gallagher D , et al. Total and regional appendicular skeletal muscle mass prediction from dual‐energy X‐ray absorptiometry body composition models. Sci Rep 2023;13:2590. 36788294 10.1038/s41598-023-29827-y PMC9929067

[102] Maughan RJ , Watson JS , Weir J . Strength and cross‐sectional area of human skeletal muscle. J Physiol 1983;338:37–49. 6875963 10.1113/jphysiol.1983.sp014658 PMC1197179

[103] Enoka RM , Duchateau J . Muscle function: strength, speed, and fatigability. In Muscle and exercise physiology. Elsevier; 2019. p 129–157 Available from: https://linkinghub.elsevier.com/retrieve/pii/B9780128145937000074

[104] Seene T , Kaasik P . Muscle weakness in the elderly: role of sarcopenia, dynapenia, and possibilities for rehabilitation. Eur Rev Aging Phys Act 2012;9:109–117.

[105] Wang Q , Jiang X , Shen Y , Yao P , Chen J , Zhou Y , et al. Effectiveness of exercise intervention on fall‐related fractures in older adults: a systematic review and meta‐analysis of randomized controlled trials. BMC Geriatr 2020;20:322. 32887571 10.1186/s12877-020-01721-6 PMC7650290

[106] Compston JE , Flahive J , Hosmer DW , Watts NB , Siris ES , Silverman S , et al. Relationship of weight, height, and body mass index with fracture risk at different sites in postmenopausal women: the Global Longitudinal study of Osteoporosis in Women (GLOW). J Bone Miner Res Off J Am Soc Bone Miner Res 2014;29:487–493. 10.1002/jbmr.2051 PMC4878680 23873741

[107] Internationale Atomenergie‐Organisation . Dual energy X ray absorptiometry for bone mineral density and body composition assessment. IAEA Human Health Series. Vienna: IAEA; 2010. 115 p.

[108] McCarthy C , Schoeller D , Brown JC , Gonzalez MC , Varanoske AN , Cataldi D , et al. D3‐creatine dilution for skeletal muscle mass measurement: historical development and current status. J Cachexia Sarcopenia Muscle 2022;13:2595–2607. 36059250 10.1002/jcsm.13083 PMC9745476

[109] Zhao R , Li X , Jiang Y , Su N , Li J , Kang L , et al. Evaluation of appendicular muscle mass in sarcopenia in older adults using ultrasonography: a systematic review and meta‐analysis. Gerontology 2022;25:1–25. 10.1159/000525758 PMC9677864 35878591

[110] Santhanam P , Nath T , Peng C , Bai H , Zhang H , Ahima RS , et al. Artificial intelligence and body composition. Diabetes Metab Syndr Clin Res Rev 2023;17:102732. 10.1016/j.dsx.2023.102732 36867973

[111] Smets J , Shevroja E , Hügle T , Leslie WD , Hans D . Machine learning solutions for osteoporosis—a review. J Bone Miner Res 2021;36:833–851. 33751686 10.1002/jbmr.4292

[112] Pickhardt PJ , Perez AA , Garrett JW , Graffy PM , Zea R , Summers RM . Fully automated deep learning tool for sarcopenia assessment on CT: L1 versus L3 vertebral level muscle measurements for opportunistic prediction of adverse clinical outcomes. AJR Am J Roentgenol 2022;218:124–131. 34406056 10.2214/AJR.21.26486 PMC9028606

[113] Janssen I , Heymsfield SB , Wang ZM , Ross R . Skeletal muscle mass and distribution in 468 men and women aged 18–88 yr. J Appl Physiol Bethesda Md 1985 2000;89:81–88. 10.1152/jappl.2000.89.1.81 10904038

[114] Fragala MS , Alley DE , Shardell MD , Harris TB , McLean RR , Kiel DP , et al. Comparison of handgrip and leg extension strength in predicting slow gait speed in older adults. J Am Geriatr Soc 2016;64:144–150. 26782864 10.1111/jgs.13871 PMC5490244

[115] Cooper R , Kuh D , Cooper C , Gale CR , Lawlor DA , Matthews F , et al. Objective measures of physical capability and subsequent health: a systematic review. Age Ageing 2011;40:14–23. 20843964 10.1093/ageing/afq117 PMC3000177

[116] Frost HM . Bone's mechanostat: a 2003 update. Anat Rec A Discov Mol Cell Evol Biol 2003;275A:1081–1101. 10.1002/ar.a.10119 14613308

[117] Heymsfield S , Prado CM , Gonzalez MC . Skeletal muscle‐focused guideline development: hierarchal model incorporating muscle form, function, and clinical outcomes. Appl Physiol Nutr Metab 2023;48:apnm‐2023‐0176. 10.1139/apnm-2023-0176 37473448

