# Supplementary material for: Muscle parameters in fragility fracture risk prediction in older adults: A scoping review
Source: J Cachexia Sarcopenia Muscle. 2024 Jan 29;15(2):477–500. doi: 10.1002/jcsm.13418 (PMC10995267; doi:10.1002/jcsm.13418)
Supplement: Supplementary file 1 — Data S1. Search equations syntaxes – Part I. Data S2. Search equations syntaxes – Part II. Table S1. Preferred Reporting Items for Systematic Reviews and Meta‐Analysis extension for Scoping Reviews (PRISMA‐ScR) checklist – Part I. Table S1. Preferred Reporting Items for Systematic Reviews and Meta‐Analysis extension for Scoping Reviews (PRISMA‐ScR) checklist – Part II. Table S2. Summary of the main analysis for each muscle assessment and each fracture types including the gaps. Figure S3f. Muscle mass parameters and risk of incident fragility fractures: All fragility fractures. Figure S4f. Muscle strength parameters and risk of incident fragility fractures: All fragility fractures. Figure S5f. Muscle function parameters and risk of incident fragility fractures: All fragility fractures. [file JCSM-15-477-s001.pptx]

## Slide 1
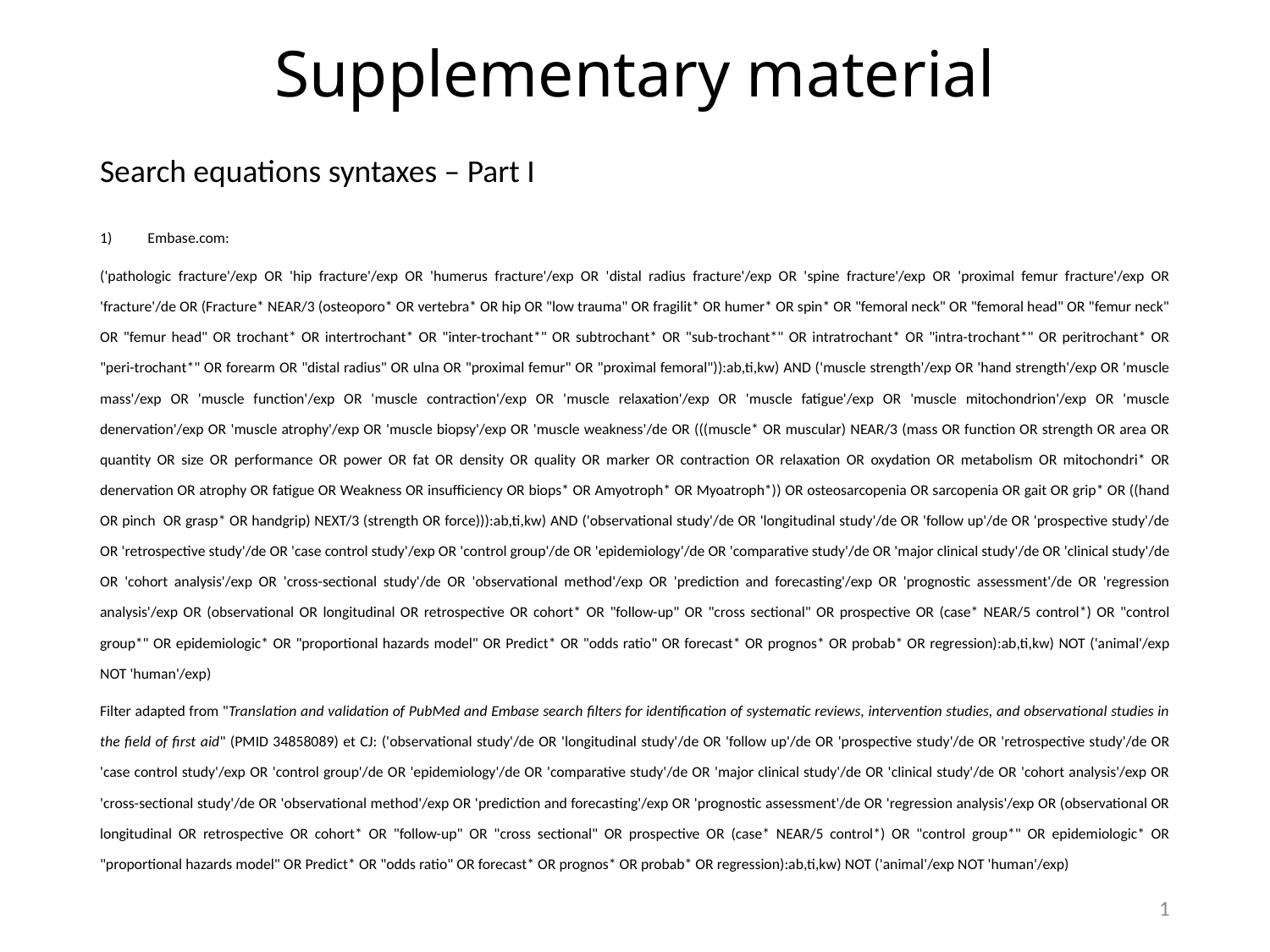

Supplementary material
# Search equations syntaxes – Part I
Embase.com:
('pathologic fracture'/exp OR 'hip fracture'/exp OR 'humerus fracture'/exp OR 'distal radius fracture'/exp OR 'spine fracture'/exp OR 'proximal femur fracture'/exp OR 'fracture'/de OR (Fracture* NEAR/3 (osteoporo* OR vertebra* OR hip OR "low trauma" OR fragilit* OR humer* OR spin* OR "femoral neck" OR "femoral head" OR "femur neck" OR "femur head" OR trochant* OR intertrochant* OR "inter-trochant*" OR subtrochant* OR "sub-trochant*" OR intratrochant* OR "intra-trochant*" OR peritrochant* OR "peri-trochant*" OR forearm OR "distal radius" OR ulna OR "proximal femur" OR "proximal femoral")):ab,ti,kw) AND ('muscle strength'/exp OR 'hand strength'/exp OR 'muscle mass'/exp OR 'muscle function'/exp OR 'muscle contraction'/exp OR 'muscle relaxation'/exp OR 'muscle fatigue'/exp OR 'muscle mitochondrion'/exp OR 'muscle denervation'/exp OR 'muscle atrophy'/exp OR 'muscle biopsy'/exp OR 'muscle weakness'/de OR (((muscle* OR muscular) NEAR/3 (mass OR function OR strength OR area OR quantity OR size OR performance OR power OR fat OR density OR quality OR marker OR contraction OR relaxation OR oxydation OR metabolism OR mitochondri* OR denervation OR atrophy OR fatigue OR Weakness OR insufficiency OR biops* OR Amyotroph* OR Myoatroph*)) OR osteosarcopenia OR sarcopenia OR gait OR grip* OR ((hand OR pinch OR grasp* OR handgrip) NEXT/3 (strength OR force))):ab,ti,kw) AND ('observational study'/de OR 'longitudinal study'/de OR 'follow up'/de OR 'prospective study'/de OR 'retrospective study'/de OR 'case control study'/exp OR 'control group'/de OR 'epidemiology'/de OR 'comparative study'/de OR 'major clinical study'/de OR 'clinical study'/de OR 'cohort analysis'/exp OR 'cross-sectional study'/de OR 'observational method'/exp OR 'prediction and forecasting'/exp OR 'prognostic assessment'/de OR 'regression analysis'/exp OR (observational OR longitudinal OR retrospective OR cohort* OR "follow-up" OR "cross sectional" OR prospective OR (case* NEAR/5 control*) OR "control group*" OR epidemiologic* OR "proportional hazards model" OR Predict* OR "odds ratio" OR forecast* OR prognos* OR probab* OR regression):ab,ti,kw) NOT ('animal'/exp NOT 'human'/exp)
Filter adapted from "Translation and validation of PubMed and Embase search filters for identification of systematic reviews, intervention studies, and observational studies in the field of first aid" (PMID 34858089) et CJ: ('observational study'/de OR 'longitudinal study'/de OR 'follow up'/de OR 'prospective study'/de OR 'retrospective study'/de OR 'case control study'/exp OR 'control group'/de OR 'epidemiology'/de OR 'comparative study'/de OR 'major clinical study'/de OR 'clinical study'/de OR 'cohort analysis'/exp OR 'cross-sectional study'/de OR 'observational method'/exp OR 'prediction and forecasting'/exp OR 'prognostic assessment'/de OR 'regression analysis'/exp OR (observational OR longitudinal OR retrospective OR cohort* OR "follow-up" OR "cross sectional" OR prospective OR (case* NEAR/5 control*) OR "control group*" OR epidemiologic* OR "proportional hazards model" OR Predict* OR "odds ratio" OR forecast* OR prognos* OR probab* OR regression):ab,ti,kw) NOT ('animal'/exp NOT 'human'/exp)
1

## Slide 2
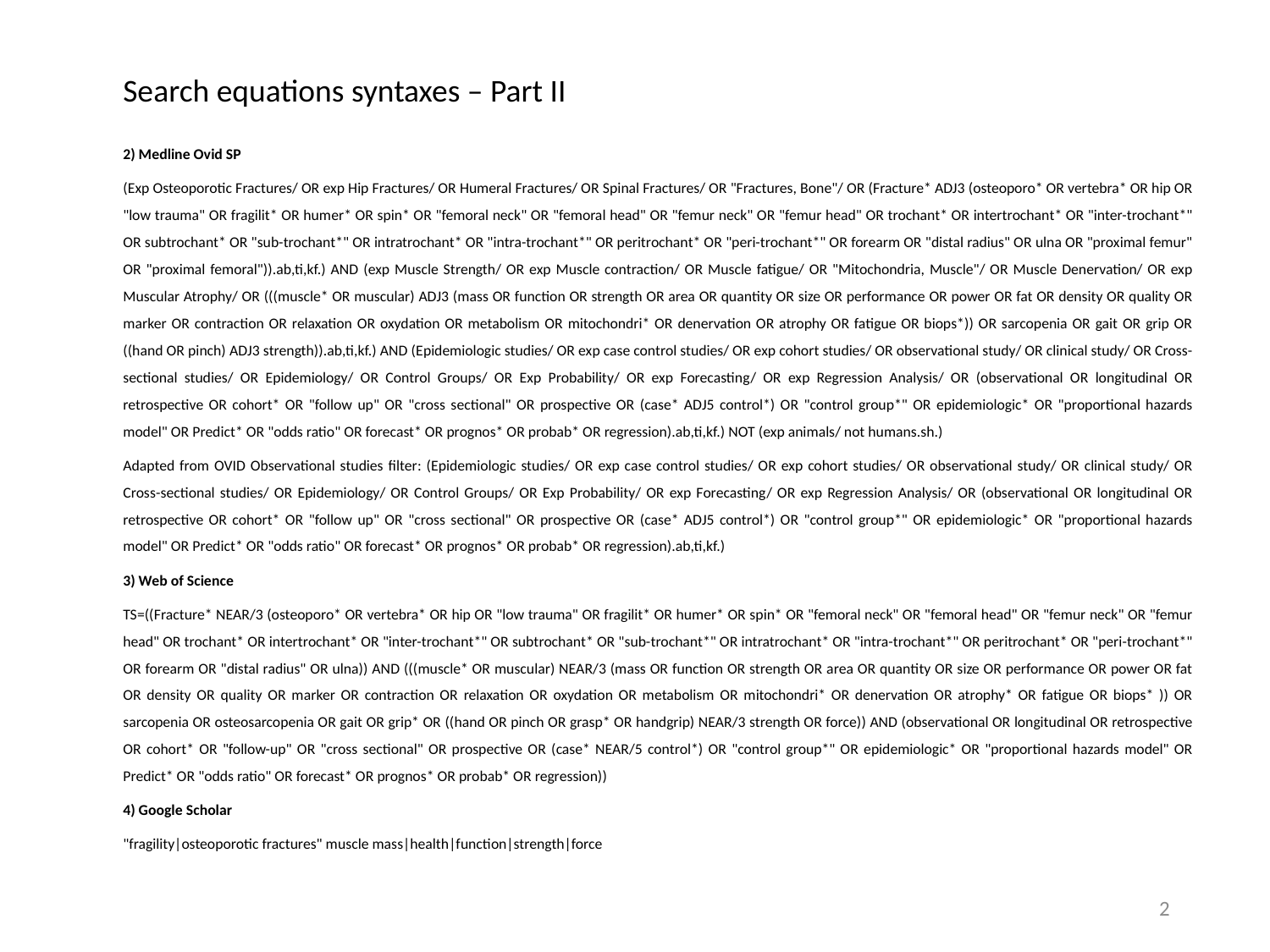

# Search equations syntaxes – Part II
2) Medline Ovid SP
(Exp Osteoporotic Fractures/ OR exp Hip Fractures/ OR Humeral Fractures/ OR Spinal Fractures/ OR "Fractures, Bone"/ OR (Fracture* ADJ3 (osteoporo* OR vertebra* OR hip OR "low trauma" OR fragilit* OR humer* OR spin* OR "femoral neck" OR "femoral head" OR "femur neck" OR "femur head" OR trochant* OR intertrochant* OR "inter-trochant*" OR subtrochant* OR "sub-trochant*" OR intratrochant* OR "intra-trochant*" OR peritrochant* OR "peri-trochant*" OR forearm OR "distal radius" OR ulna OR "proximal femur" OR "proximal femoral")).ab,ti,kf.) AND (exp Muscle Strength/ OR exp Muscle contraction/ OR Muscle fatigue/ OR "Mitochondria, Muscle"/ OR Muscle Denervation/ OR exp Muscular Atrophy/ OR (((muscle* OR muscular) ADJ3 (mass OR function OR strength OR area OR quantity OR size OR performance OR power OR fat OR density OR quality OR marker OR contraction OR relaxation OR oxydation OR metabolism OR mitochondri* OR denervation OR atrophy OR fatigue OR biops*)) OR sarcopenia OR gait OR grip OR ((hand OR pinch) ADJ3 strength)).ab,ti,kf.) AND (Epidemiologic studies/ OR exp case control studies/ OR exp cohort studies/ OR observational study/ OR clinical study/ OR Cross-sectional studies/ OR Epidemiology/ OR Control Groups/ OR Exp Probability/ OR exp Forecasting/ OR exp Regression Analysis/ OR (observational OR longitudinal OR retrospective OR cohort* OR "follow up" OR "cross sectional" OR prospective OR (case* ADJ5 control*) OR "control group*" OR epidemiologic* OR "proportional hazards model" OR Predict* OR "odds ratio" OR forecast* OR prognos* OR probab* OR regression).ab,ti,kf.) NOT (exp animals/ not humans.sh.)
Adapted from OVID Observational studies filter: (Epidemiologic studies/ OR exp case control studies/ OR exp cohort studies/ OR observational study/ OR clinical study/ OR Cross-sectional studies/ OR Epidemiology/ OR Control Groups/ OR Exp Probability/ OR exp Forecasting/ OR exp Regression Analysis/ OR (observational OR longitudinal OR retrospective OR cohort* OR "follow up" OR "cross sectional" OR prospective OR (case* ADJ5 control*) OR "control group*" OR epidemiologic* OR "proportional hazards model" OR Predict* OR "odds ratio" OR forecast* OR prognos* OR probab* OR regression).ab,ti,kf.)
3) Web of Science
TS=((Fracture* NEAR/3 (osteoporo* OR vertebra* OR hip OR "low trauma" OR fragilit* OR humer* OR spin* OR "femoral neck" OR "femoral head" OR "femur neck" OR "femur head" OR trochant* OR intertrochant* OR "inter-trochant*" OR subtrochant* OR "sub-trochant*" OR intratrochant* OR "intra-trochant*" OR peritrochant* OR "peri-trochant*" OR forearm OR "distal radius" OR ulna)) AND (((muscle* OR muscular) NEAR/3 (mass OR function OR strength OR area OR quantity OR size OR performance OR power OR fat OR density OR quality OR marker OR contraction OR relaxation OR oxydation OR metabolism OR mitochondri* OR denervation OR atrophy* OR fatigue OR biops* )) OR sarcopenia OR osteosarcopenia OR gait OR grip* OR ((hand OR pinch OR grasp* OR handgrip) NEAR/3 strength OR force)) AND (observational OR longitudinal OR retrospective OR cohort* OR "follow-up" OR "cross sectional" OR prospective OR (case* NEAR/5 control*) OR "control group*" OR epidemiologic* OR "proportional hazards model" OR Predict* OR "odds ratio" OR forecast* OR prognos* OR probab* OR regression))
4) Google Scholar
"fragility|osteoporotic fractures" muscle mass|health|function|strength|force
2

## Slide 3
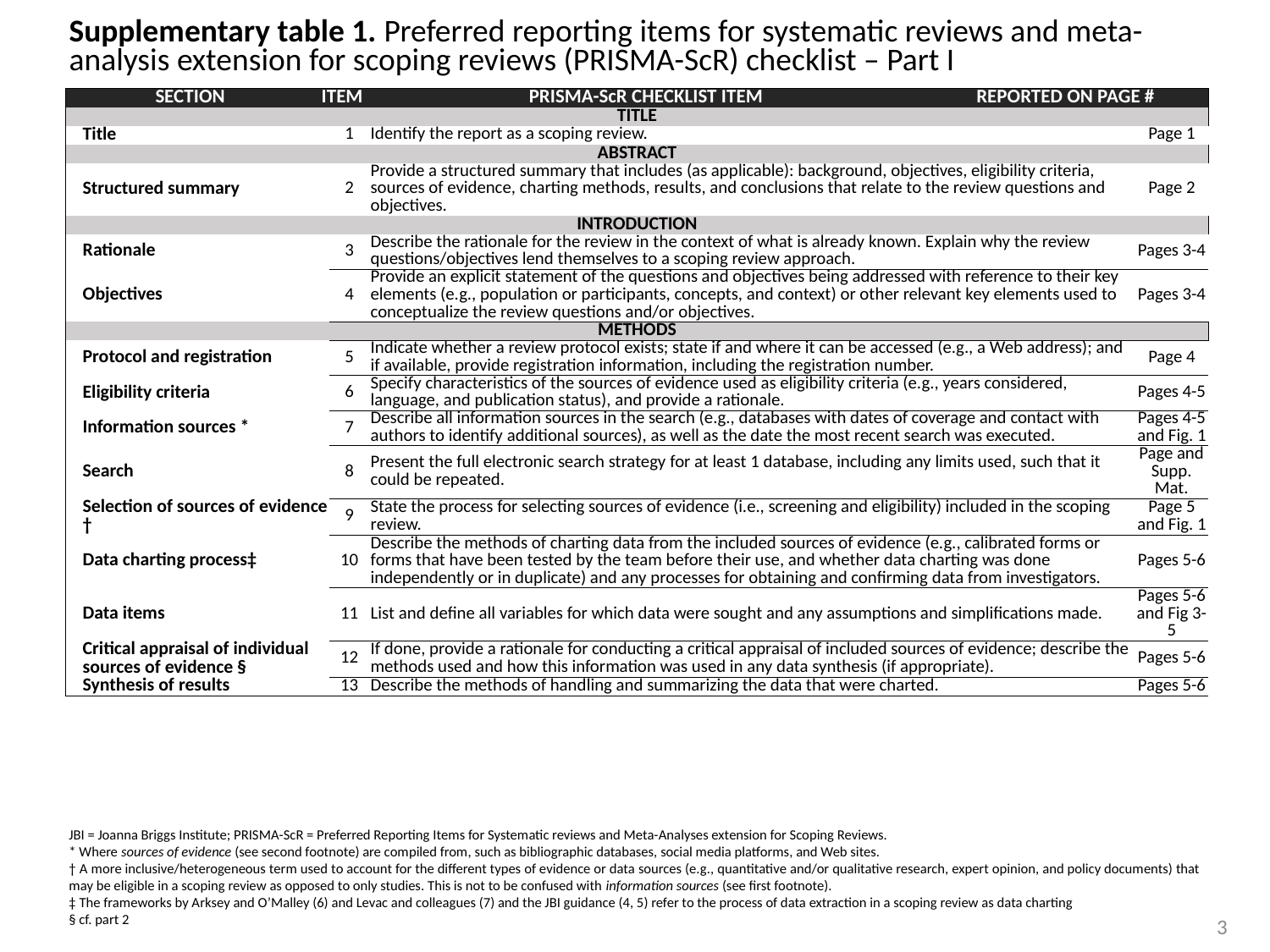

Supplementary table 1. Preferred reporting items for systematic reviews and meta-analysis extension for scoping reviews (PRISMA-ScR) checklist – Part I
| SECTION | ITEM | ITEM | PRISMA-ScR CHECKLIST ITEM | REPORTED ON PAGE # | |
| --- | --- | --- | --- | --- | --- |
| TITLE | | | | | |
| Title | | 1 | Identify the report as a scoping review. | 1 | Page 1 |
| ABSTRACT | | | | | |
| Structured summary | | 2 | Provide a structured summary that includes (as applicable): background, objectives, eligibility criteria, sources of evidence, charting methods, results, and conclusions that relate to the review questions and objectives. | 2 | Page 2 |
| INTRODUCTION | | | | | |
| Rationale | | 3 | Describe the rationale for the review in the context of what is already known. Explain why the review questions/objectives lend themselves to a scoping review approach. | 4 | Pages 3-4 |
| Objectives | | 4 | Provide an explicit statement of the questions and objectives being addressed with reference to their key elements (e.g., population or participants, concepts, and context) or other relevant key elements used to conceptualize the review questions and/or objectives. | 4-5 | Pages 3-4 |
| METHODS | | | | | |
| Protocol and registration | | 5 | Indicate whether a review protocol exists; state if and where it can be accessed (e.g., a Web address); and if available, provide registration information, including the registration number. | 4-5 | Page 4 |
| Eligibility criteria | | 6 | Specify characteristics of the sources of evidence used as eligibility criteria (e.g., years considered, language, and publication status), and provide a rationale. | 5 | Pages 4-5 |
| Information sources \* | | 7 | Describe all information sources in the search (e.g., databases with dates of coverage and contact with authors to identify additional sources), as well as the date the most recent search was executed. | 5 | Pages 4-5 and Fig. 1 |
| Search | | 8 | Present the full electronic search strategy for at least 1 database, including any limits used, such that it could be repeated. | 5 | Page and Supp. Mat. |
| Selection of sources of evidence † | | 9 | State the process for selecting sources of evidence (i.e., screening and eligibility) included in the scoping review. | 5 | Page 5 and Fig. 1 |
| Data charting process‡ | | 10 | Describe the methods of charting data from the included sources of evidence (e.g., calibrated forms or forms that have been tested by the team before their use, and whether data charting was done independently or in duplicate) and any processes for obtaining and confirming data from investigators. | 5-6 | Pages 5-6 |
| Data items | | 11 | List and define all variables for which data were sought and any assumptions and simplifications made. | 5-6 Cf. fig 3-5 | Pages 5-6 and Fig 3-5 |
| Critical appraisal of individual sources of evidence § | | 12 | If done, provide a rationale for conducting a critical appraisal of included sources of evidence; describe the methods used and how this information was used in any data synthesis (if appropriate). | 5-6 | Pages 5-6 |
| Synthesis of results | | 13 | Describe the methods of handling and summarizing the data that were charted. | 5-6 | Pages 5-6 |
JBI = Joanna Briggs Institute; PRISMA-ScR = Preferred Reporting Items for Systematic reviews and Meta-Analyses extension for Scoping Reviews.
* Where sources of evidence (see second footnote) are compiled from, such as bibliographic databases, social media platforms, and Web sites.
† A more inclusive/heterogeneous term used to account for the different types of evidence or data sources (e.g., quantitative and/or qualitative research, expert opinion, and policy documents) that may be eligible in a scoping review as opposed to only studies. This is not to be confused with information sources (see first footnote).
‡ The frameworks by Arksey and O’Malley (6) and Levac and colleagues (7) and the JBI guidance (4, 5) refer to the process of data extraction in a scoping review as data charting
§ cf. part 2
3

## Slide 4
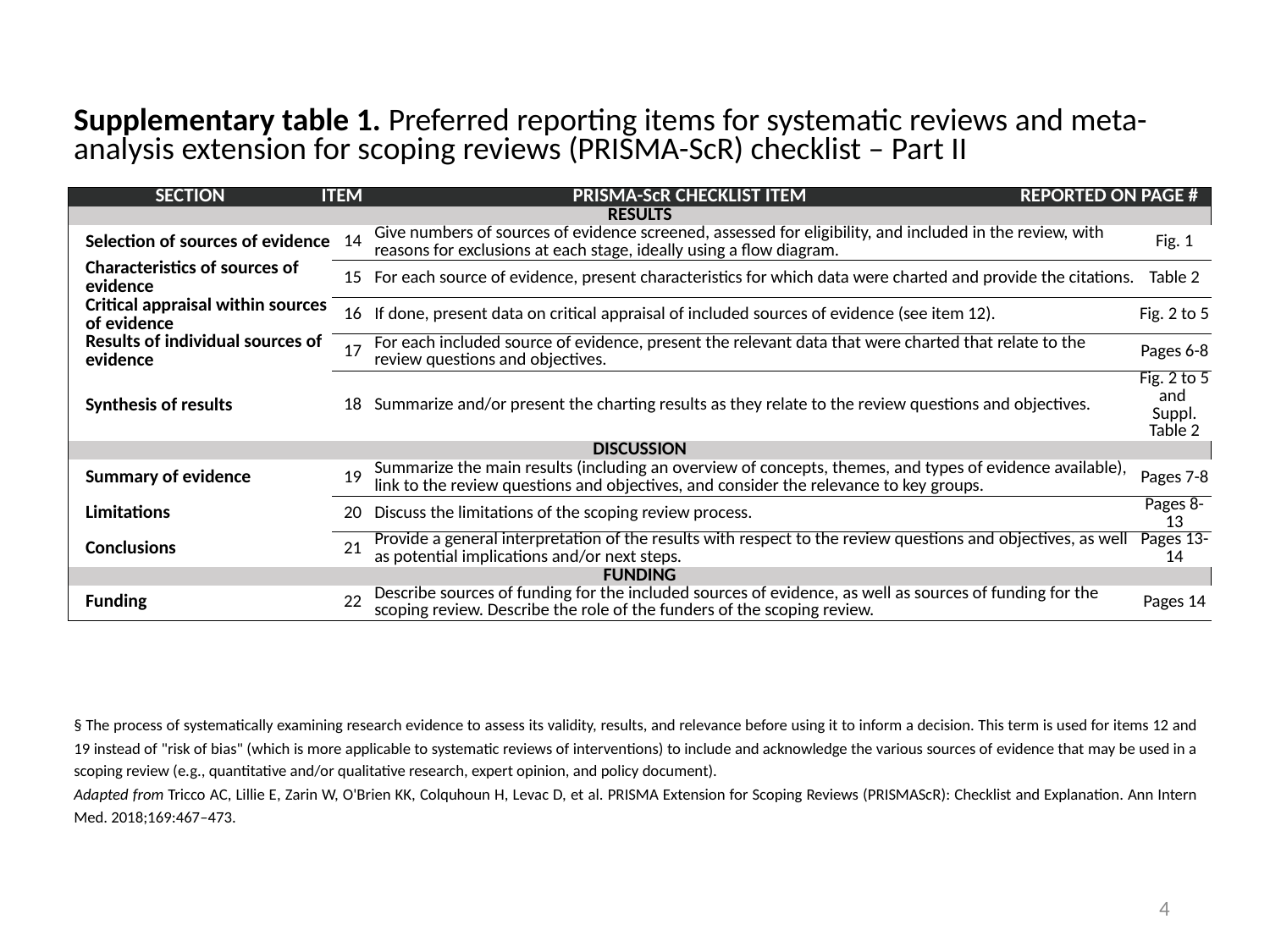

Supplementary table 1. Preferred reporting items for systematic reviews and meta-analysis extension for scoping reviews (PRISMA-ScR) checklist – Part II
| SECTION | ITEM | ITEM | PRISMA-ScR CHECKLIST ITEM | REPORTED ON PAGE # | PRISMA-ScR CHECKLIST ITEM |
| --- | --- | --- | --- | --- | --- |
| RESULTS | | | | | |
| Selection of sources of evidence | | 14 | Give numbers of sources of evidence screened, assessed for eligibility, and included in the review, with reasons for exclusions at each stage, ideally using a flow diagram. | | Fig. 1 |
| Characteristics of sources of evidence | | 15 | For each source of evidence, present characteristics for which data were charted and provide the citations. | | Table 2 |
| Critical appraisal within sources of evidence | | 16 | If done, present data on critical appraisal of included sources of evidence (see item 12). | | Fig. 2 to 5 |
| Results of individual sources of evidence | | 17 | For each included source of evidence, present the relevant data that were charted that relate to the review questions and objectives. | | Pages 6-8 |
| Synthesis of results | | 18 | Summarize and/or present the charting results as they relate to the review questions and objectives. | | Fig. 2 to 5 and Suppl. Table 2 |
| DISCUSSION | | | | | |
| Summary of evidence | | 19 | Summarize the main results (including an overview of concepts, themes, and types of evidence available), link to the review questions and objectives, and consider the relevance to key groups. | | Pages 7-8 |
| Limitations | | 20 | Discuss the limitations of the scoping review process. | | Pages 8-13 |
| Conclusions | | 21 | Provide a general interpretation of the results with respect to the review questions and objectives, as well as potential implications and/or next steps. | | Pages 13-14 |
| FUNDING | | | | | |
| Funding | | 22 | Describe sources of funding for the included sources of evidence, as well as sources of funding for the scoping review. Describe the role of the funders of the scoping review. | | Pages 14 |
§ The process of systematically examining research evidence to assess its validity, results, and relevance before using it to inform a decision. This term is used for items 12 and 19 instead of "risk of bias" (which is more applicable to systematic reviews of interventions) to include and acknowledge the various sources of evidence that may be used in a scoping review (e.g., quantitative and/or qualitative research, expert opinion, and policy document).
Adapted from Tricco AC, Lillie E, Zarin W, O'Brien KK, Colquhoun H, Levac D, et al. PRISMA Extension for Scoping Reviews (PRISMAScR): Checklist and Explanation. Ann Intern Med. 2018;169:467–473.
4

## Slide 5
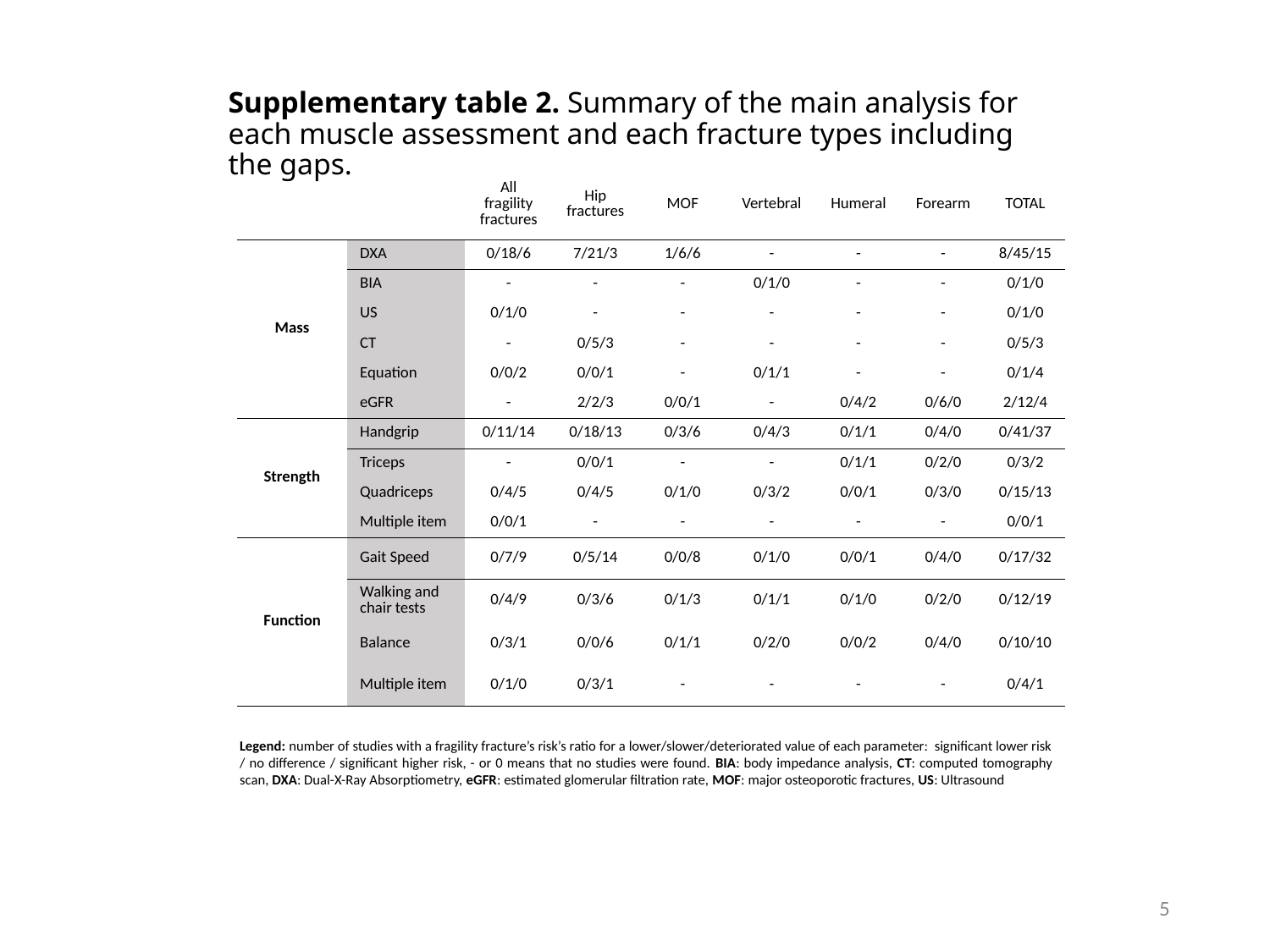

Supplementary table 2. Summary of the main analysis for each muscle assessment and each fracture types including the gaps.
| | Muscle Mass | All fragility fractures | Hip fractures | MOF | Vertebral | Humeral | Forearm | TOTAL |
| --- | --- | --- | --- | --- | --- | --- | --- | --- |
| Mass | DXA | 0/18/6 | 7/21/3 | 1/6/6 | - | - | - | 8/45/15 |
| | BIA | - | - | - | 0/1/0 | - | - | 0/1/0 |
| | US | 0/1/0 | - | - | - | - | - | 0/1/0 |
| | CT | - | 0/5/3 | - | - | - | - | 0/5/3 |
| | Equation | 0/0/2 | 0/0/1 | - | 0/1/1 | - | - | 0/1/4 |
| | eGFR | - | 2/2/3 | 0/0/1 | - | 0/4/2 | 0/6/0 | 2/12/4 |
| Strength | Handgrip | 0/11/14 | 0/18/13 | 0/3/6 | 0/4/3 | 0/1/1 | 0/4/0 | 0/41/37 |
| | Triceps | - | 0/0/1 | - | - | 0/1/1 | 0/2/0 | 0/3/2 |
| | Quadriceps | 0/4/5 | 0/4/5 | 0/1/0 | 0/3/2 | 0/0/1 | 0/3/0 | 0/15/13 |
| | Multiple item | 0/0/1 | - | - | - | - | - | 0/0/1 |
| Function | Gait Speed | 0/7/9 | 0/5/14 | 0/0/8 | 0/1/0 | 0/0/1 | 0/4/0 | 0/17/32 |
| | Walking and chair tests | 0/4/9 | 0/3/6 | 0/1/3 | 0/1/1 | 0/1/0 | 0/2/0 | 0/12/19 |
| | Balance | 0/3/1 | 0/0/6 | 0/1/1 | 0/2/0 | 0/0/2 | 0/4/0 | 0/10/10 |
| | Multiple item | 0/1/0 | 0/3/1 | - | - | - | - | 0/4/1 |
Legend: number of studies with a fragility fracture’s risk’s ratio for a lower/slower/deteriorated value of each parameter: significant lower risk / no difference / significant higher risk, - or 0 means that no studies were found. BIA: body impedance analysis, CT: computed tomography scan, DXA: Dual-X-Ray Absorptiometry, eGFR: estimated glomerular filtration rate, MOF: major osteoporotic fractures, US: Ultrasound
5

## Slide 6
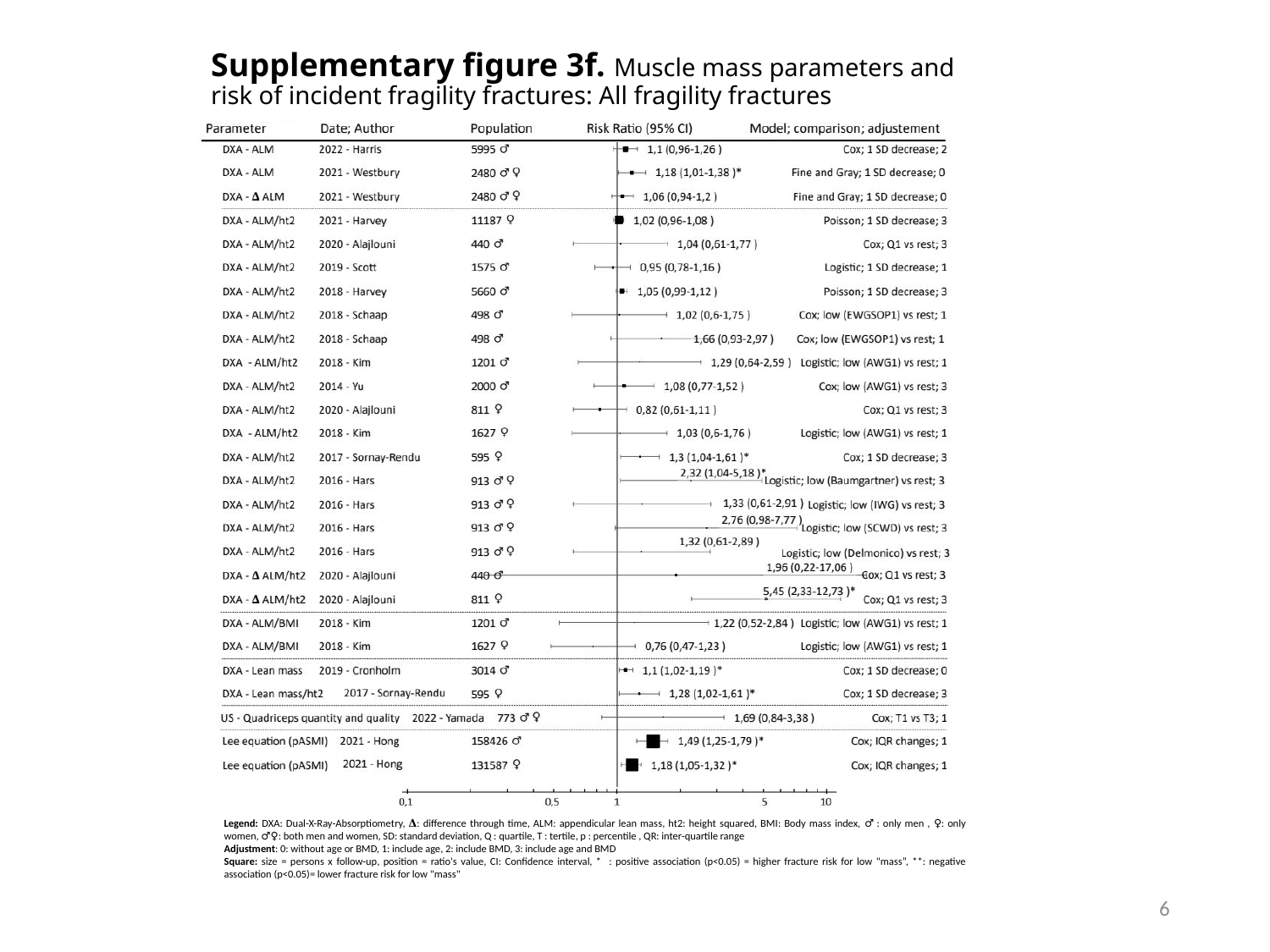

# Supplementary figure 3f. Muscle mass parameters and risk of incident fragility fractures: All fragility fractures
Legend: DXA: Dual-X-Ray-Absorptiometry, 𝚫: difference through time, ALM: appendicular lean mass, ht2: height squared, BMI: Body mass index, ♂ : only men , ♀: only women, ♂♀: both men and women, SD: standard deviation, Q : quartile, T : tertile, p : percentile , QR: inter-quartile range
Adjustment: 0: without age or BMD, 1: include age, 2: include BMD, 3: include age and BMD
Square: size = persons x follow-up, position = ratio's value, CI: Confidence interval, * : positive association (p<0.05) = higher fracture risk for low "mass”, **: negative association (p<0.05)= lower fracture risk for low "mass"
6

## Slide 7
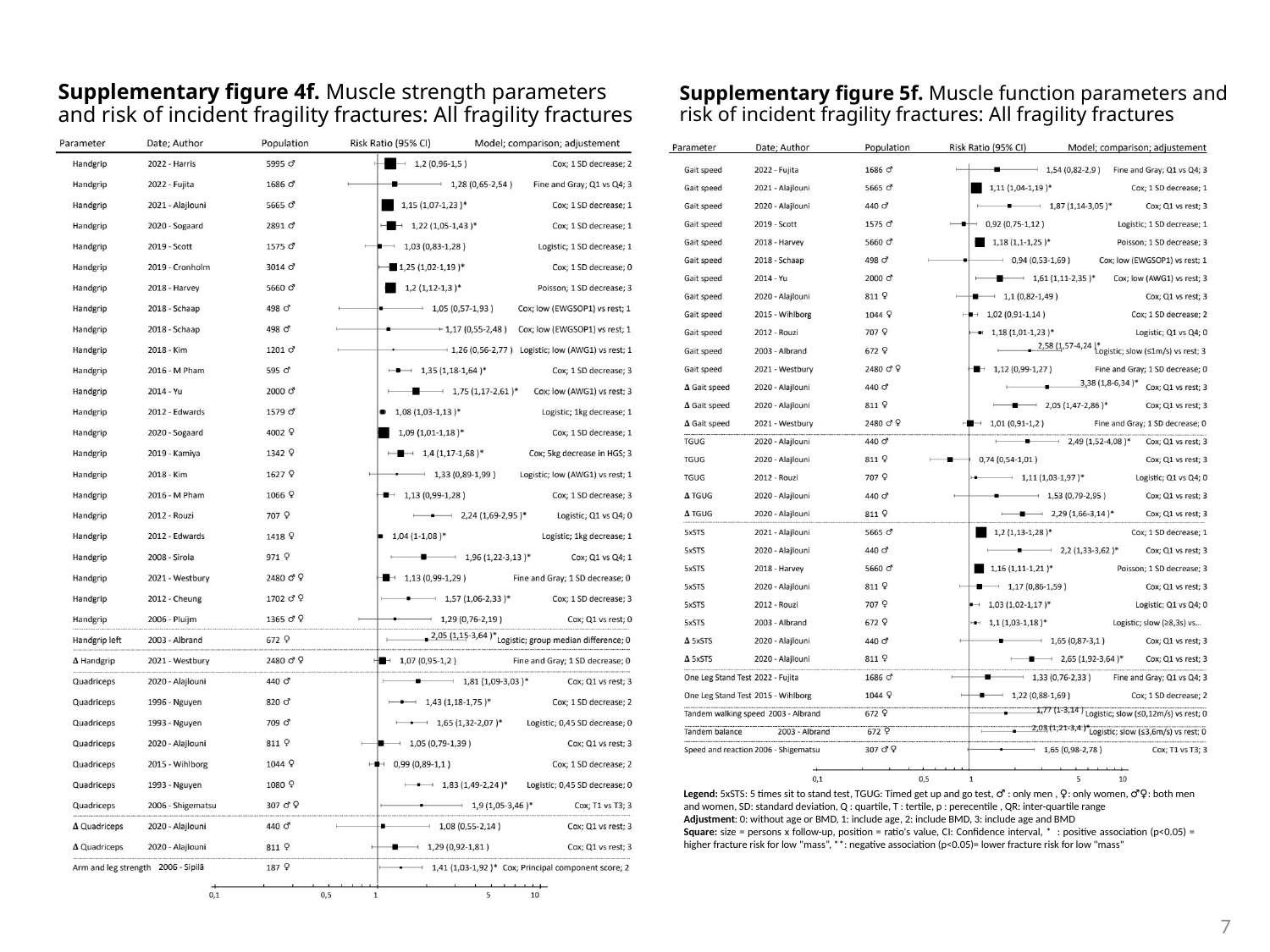

Supplementary figure 4f. Muscle strength parameters and risk of incident fragility fractures: All fragility fractures
Supplementary figure 5f. Muscle function parameters and risk of incident fragility fractures: All fragility fractures
Legend: 5xSTS: 5 times sit to stand test, TGUG: Timed get up and go test, ♂ : only men , ♀: only women, ♂♀: both men and women, SD: standard deviation, Q : quartile, T : tertile, p : perecentile , QR: inter-quartile range
Adjustment: 0: without age or BMD, 1: include age, 2: include BMD, 3: include age and BMD
Square: size = persons x follow-up, position = ratio's value, CI: Confidence interval, * : positive association (p<0.05) = higher fracture risk for low "mass”, **: negative association (p<0.05)= lower fracture risk for low "mass"
7
